# TM9SF1 drives the lipophagic flux via AMPK-ULK1 signaling to sustain metabolic fitness in HER2-positive breast cancer

**DOI:** 10.1038/s41419-025-08093-y

**Published:** 2025-10-24

**Authors:** Xiaofen Li, Xiaoqin Yu, Kaiyan Huang, Xin Yu, Shiping Luo, Xiewei Huang, Chuangui Song

**Affiliations:** 1 Department of Breast Surgery, Clinical Oncology School of Fujian Medical University, Fujian Cancer Hospital (Fujian Branch of Fudan University Shanghai Cancer Center), Fuzhou, China; 2 Fujian Medical University, Fuzhou, China; 3 Department of Breast Surgery, The Second Affiliated Hospital of Fujian Medical University, Quanzhou, China

**Keywords:** Breast cancer, Prognostic markers, Autophagy

## Abstract

Therapeutic resistance and recurrence in human epidermal growth factor receptor 2-positive breast cancer (HER2 + BC) remain critical challenges that portend poor patient outcomes. Dysregulated autophagy and lipid metabolism contribute to tumor progression, yet the crosstalk between these pathways is poorly understood. This study investigates the role of transmembrane 9 superfamily member 1 (TM9SF1) in lipophagy and lipid metabolic reprogramming in HER2 + BC under metabolic stress. Clinically, TM9SF1 was significantly upregulated in HER2 + BC tissues and correlated with poor prognosis. Functionally, its expression correlated with markers of enhanced autophagy and lysosomal lipid catabolism, and it promoted tumor cell proliferation in vitro and in vivo. Conversely, TM9SF1 knockdown suppressed lipophagy under both basal and starvation conditions, inhibiting lipid droplet (LD) hydrolysis and the conversion of triglycerides to free fatty acids. This suppression was phenotypically characterized by LD accumulation, reduced autophagosomes and lipophagosomes, and altered enzymatic and lipidomic profiles. Mechanistically, TM9SF1 sustained lipophagy by promoting the phosphorylation of AMP-activated protein kinase at Thr172 and UNC-51-like kinase 1 at Ser555. Consequently, TM9SF1 was pivotal for lipid metabolic reprogramming, maintaining energy homeostasis and enhancing adaptation to nutrient deprivation through lipophagy. Overall, our findings identify TM9SF1 as a key HER2 + BC-associated regulator that drives lipophagy via the AMP-activated protein kinase-UNC-51-like kinase 1 pathway, facilitating LD turnover and free fatty acids utilization to sustain energy homeostasis in HER2 + BC. This work establishes a critical link between malignant phenotypes and metabolic resilience. Targeting this regulatory network represents a promising strategy to dismantle the metabolic scaffolds underlying HER2 + BC aggressiveness and therapeutic resistance.

## Introduction

Human epidermal growth factor receptor 2 (HER2)-positive breast cancer (HER2 + BC) accounts for approximately 15–20% of all breast cancer cases. HER2 overexpression drives aberrant cancer cell proliferation and promotes malignant progression, leading to faster disease advancement and worse prognosis in patients with HER2 + BC than in those with HER2-negative tumors [1–3]. Although targeted therapies, such as trastuzumab, have improved clinical outcomes, therapeutic resistance remains a major clinical hurdle [4–6]. This resistance is increasingly linked to metabolic adaptation, particularly the reprogramming of lipid metabolism to fuel energy production and membrane synthesis [7].

Autophagy is a lysosome-dependent catabolic process that enables cancer cells to survive metabolic stress by degrading cytoplasmic components into reusable metabolites [8–11]. Among its specialized forms [12, 13], lipophagy—the selective autophagic degradation of lipid droplets (LDs)—serves as a critical nexus between autophagy and lipid catabolism. Unlike canonical lipolysis mediated by cytosolic lipases (e.g., adipose triglyceride lipase), lipophagy delivers LDs to lysosomes for hydrolysis by lysosomal acid lipase (LIPA), releasing free fatty acids (FFAs) for mitochondrial β-oxidation [14, 15]. This dual reliance on autophagic machinery and lipid breakdown endows cancer cells with the metabolic flexibility to dynamically balance energy storage and utilization under fluctuating nutrient conditions, a capability that remains underexplored in HER2 + BC [16–18].

Transmembrane 9 superfamily member 1 (TM9SF1) has recently emerged as a modulator of autophagy and a potential prognostic marker in several cancers [19–23]. However, the role of TM9SF1 in coordinating the interplay between autophagy and lipid catabolism, particularly in HER2 + BC, remains unknown. The AMP-activated protein kinase (AMPK)-UNC-51-like kinase 1 (ULK1) pathway is a central regulator coupling cellular energy stress with autophagosome formation [24–26]. Under low-energy conditions, activated AMPK phosphorylates ULK1 to initiate autophagy and lipophagy, thereby degrading LDs to replenish cellular energy pools [27]. We hypothesize that TM9SF1 orchestrates autophagic activation with LD degradation through this regulatory network, enabling HER2 + BC to exploit lipophagy for metabolic resilience.

Herein, we identify TM9SF1 as a critical metabolic nexus in HER2 + BC that governs lipophagy through AMPK-ULK1 signaling. By dissecting how TM9SF1-mediated LD turnover sustains energy homeostasis under nutrient deprivation, this study illuminates a convergence point between malignant tumor phenotypes and lipid metabolic fitness, with profound therapeutic implications for overcoming resistance in HER2 + BC.

## Methods

### Clinical samples and ethics statement

Transcriptomic data (RNA-seq) for all available 180 female HER2 + BC patients and 22 normal controls were obtained from The Cancer Genome Atlas-Breast Cancer (TCGA-BRCA) database [28]. Additionally, a cohort of 78 HER2 + BC specimens was consecutively collected at the Fujian Cancer Hospital during our study period, of which 63 were paired with adjacent normal tissues (2 cm periphery of resected tumors). For human specimens, inclusion criteria were female patients with a confirmed diagnosis of HER2+ breast cancer as defined by an immunohistochemistry score of 3+ or a fluorescence in situ hybridization score ≥ 2.0. No pre-established exclusion criteria were applied, and no samples were excluded from the analysis during the study. The study was approved by the Ethics Committee of Fujian Cancer Hospital (K2024-521-01), and informed consent was obtained from all participants.

### Bioinformatics analysis

TCGA gene expression data were analyzed for differential expression using the ‘limma’ R package. Gene set enrichment analysis (GSEA) was performed using the Molecular Signatures Database to identify enriched pathways [29]. The prognostic significance of *TM9SF1* in HER2 + BC was assessed using the Kaplan-Meier plotter (http://kmplot.com/analysis/).

### Cell culture and stable cell line generation

MCF10A, MDA-MB-361, SKBR3, and HEK293T cells were sourced from the Cell Bank of the Type Culture Collection of the Chinese Academy of Sciences, and JIMT1 and AU565 cells were acquired from ProCell (Wuhan, China). The MCF10A cells were cultured in a specific medium (ProCell, CM-0525). SKBR3, HEK293T, JIMT1, and AU565 cells were cultured in Dulbecco’s modified Eagle medium (ProCell, PM153312) with 10% fetal bovine serum (ProCell, 164210) at 37 °C with 5% CO_2_, and MDA-MB-361 cells were cultured in Leibovitz's L-15 (ProCell, PM151010) medium with 20% fetal bovine serum (ProCell, 164210) at 37 °C with 100% air. All cells were authenticated by STR and tested for mycoplasma. Earle’s balanced salt solution (EBSS) was customized by ProCell (PB180337).

For stable cell line generation, lentiviral vectors encoding specific shRNAs (Supplementary Table S1) or the TM9SF1 coding sequence (GeneChem) were produced in HEK293T cells. Target cells were transduced and selected with puromycin for 7–10 days. Knockdown or overexpression efficiency was validated by real-time quantitative PCR (RT-qPCR) and western blotting (WB).

### RNA extraction and RT-qPCR

Total RNA was extracted using TRIzol reagent (Invitrogen, 10296-010). First-strand cDNA was synthesized using a reverse transcription kit (ABM, G592). RT-qPCR was performed using the SYBR Green Mix (Vazyme, Q111) on a real-time PCR system (Thermo Fisher Scientific, 4351107). GAPDH served as an endogenous control. Primer sequences are presented in Supplementary Table S2. Relative gene expression was calculated using the ΔΔCt method.

### WB

Cells were lysed in RIPA buffer (Beyotime, P0013B) with protease (Beyotime, P1005) and phosphatase inhibitors (Beyotime, P1082). Equal amounts of protein (30 µg) were resolved using sodium dodecyl sulfate-polyacrylamide gel electrophoresis and transferred onto a polyvinylidene fluoride membrane. Membranes were blocked with 5% non-fat milk and incubated with primary antibodies at 4 °C overnight, followed by incubation with horseradish peroxidase-conjugated-tagged secondary antibodies. ECL (Biosharp, BL520B) was used to detect protein bands. A list of the antibodies used is provided in Supplementary Table S3. Unedited WB images are shown in Supplementary Information.

### Cell proliferation and viability assays

Cell proliferation was assessed using the cell counting kit 8 (CCK8) assay, colony formation assay, and 5-ethynyl-2’-deoxyuridine (EdU) incorporation assay. For CCK8, cells were seeded at 3000 cells/well in 96-well plates. Cells were assayed using 10 µL of CCK8 (MCE, HY-K0301) for 2 h at 37 °C. The absorbance was measured at 450 nm. For colony formation, 1000 cells/well were seeded in 6-well plates and cultured for 14-20 days and followed by staining with a crystal violet solution (Beyotime, C0121) for 15 min. For EdU assays, cells were incubated with 10 µM EdU (Beyotime, C0078S) for 2 h before detection. EdU incorporation was quantified as a percentage of total Hoechst-stained cells. All assays were performed according to the manufacturer’s instructions.

### Fluorescence microscopy and staining

For visualization of lysosomes and LDs, cells were co-stained with LysoTracker Red (Beyotime, C1046) (1:10000) and BODIPY 493/503 (Beyotime, C2053S) (1×) for 30 min at room temperature. For LD quantification, cells were stained with Nile Red (1:1000) (Beyotime, C2051S) for 15 min in the dark or Oil Red O solution (Beyotime, C0158S) for 20 min at room temperature. For autophagosome detection, cells were stained with 0.1 μM Monodansylcadaverine (MDC)- propidium iodide (PI) (Beyotime, C3019S) for 30 min at 37 °C. Images were acquired on a laser scanning confocal or fluorescence microscope and analyzed using ImageJ.

### Immunohistochemistry

Paraffin-embedded tumor sections were deparaffinized, subjected to heat-induced antigen retrieval in EDTA buffer (MXB, MVS-0099), and blocked with 5% bovine serum albumin for 60 min. Sections were incubated with primary antibody at 4 °C overnight, followed by a horseradish peroxidase-conjugated secondary antibody and visualization with a DAB (MXB, DAB-0031) substrate kit. Nuclei were counterstained with hematoxylin. The expression levels of the proteins of interest were assessed by quantifying the intensity and distribution of staining within tumor sections.

### In vivo tumor formation

The sample size (*n* = 5 per group) aligns with widely accepted standards for preliminary xenograft studies, ensuring statistical feasibility while adhering to the 3 R principles (reduction, refinement, and replacement). The allocation of animals to experimental groups was randomized, and the investigators were not blinded to group allocation during the experiment or outcome assessment. Cells (5 × 10^6^) with genetic modification were suspended in 100 µL PBS and randomly injected subcutaneously into the mammary fat pad of five-week-old BALB/c female nude mice (GemPharmatech). Tumor volumes were measured every 3–4 days using the formula: volume = (length × width^2^)/2. At the end of the study, tumors were excised and weighed. All animal experiments were approved by the Ethics Committee of Fujian Medical University (IACUC FJMU 2024-0145).

### Transmission electron microscopy (TEM)

Cells were fixed with 2.5% glutaraldehyde at 4 °C for 2 h, followed by 1% osmium tetroxide post-fixation for 1 h. Cells were dehydrated using a graded ethanol series, embedded in epoxy resin, and then cut into 50 nm sections. These sections were stained with uranyl acetate and lead citrate for 15 min. The sections were examined using TEM to identify autophagosomes based on their characteristic double-membrane structures.

### Co-immunoprecipitation assays

Cells were placed in lysis buffer supplemented with protease inhibitors on ice for 30 min. The lysate was centrifuged (14,000×*g*, 15 min, 4 °C), and then pre-cleared with Protein A/G magnetic beads (MCE, #HY-K0202) for 1 h at 4 °C. Subsequently, 1–5 µg of target-specific antibody or isotype-matched control IgG was added and incubated overnight at 4 °C, followed by incubation with Protein A/G beads for 2 h. The beads were pelleted, washed with buffer, and eluted with 2× sodium dodecyl sulfate-polyacrylamide gel electrophoresis loading buffer at room temperature. Protein complexes were analyzed by WB.

### Quantitative lipidomics analysis using liquid chromatography-tandem mass spectrometry

Lipid extracts were analyzed using a UPLC system coupled with tandem mass spectrometry [30]. Chromatographic separation was performed using a Thermo Accucore C30 column with a gradient elution. Lipid quantification was performed using the Multiple Reaction Monitoring mode of a triple quadrupole mass spectrometer.

### Seahorse long-chain fatty acid oxidation (FAO) stress test

Cells were cultured at 3 × 10^4^ per well in XF24 plates and then washed with Seahorse XF medium (Agilent, 103672-100) and equilibrated in a CO_2_-free incubator for 60 min. The oxygen consumption rate was determined using a Seahorse XFe24 Analyzer, with etomoxir added, to evaluate the impact of FAO on respiration [31].

### ATP detection assay

Cells were lysed in ATP extraction buffer on ice for 10–15 min, followed by centrifugation at 12,000×*g* for 10 min at 4 °C. ATP levels were quantified using an ATP assay kit (Solarbio, BC0305).

### FFA detection assay

An assay kit (Solarbio, BC0595) was used to extract FFA. Absorbance was measured at 550 nm, and a standard curve was used to determine the FFA content in the samples.

### Quantitative proteomics analysis

For quantitative proteomics, 100 µg of protein lysate from each group was reduced with 5 mM DTT, alkylated with 11 mM iodoacetamide, and digested overnight with trypsin (Promega). The resulting peptides were desalted using C18 resin (Millipore) and separated on a Vanquish Neo UHPLC system. Data-independent acquisition mass spectrometry was performed on an Orbitrap Astral mass spectrometer (Thermo Fisher Scientific). Raw files were analyzed using DIA-NN v1.8.1 against the UniProt human database (1% false discovery rate). Proteins with a fold change ≥ 1.5 and a *p*-value < 0.05 were considered differentially expressed and were subjected to Kyoto Encyclopedia of Genes and Genomes (KEGG) pathway enrichment analysis.

### Statistical analysis

Data are presented as mean ± standard error of the mean (SEM). Unless otherwise specified, all experiments were repeated three times independently. Statistical comparisons between two groups were performed using a two-tailed Student’s *t*-test or Mann–Whitney *U*-test. Comparisons among multiple groups were performed using one-way ANOVA followed by an appropriate post-hoc test. A *p*-value < 0.05 was considered statistically significant (^*^p < 0.05, ^**^p < 0.01, ^***^p < 0.001).

## Results

### Enhanced lipophagy flux is a metabolic signature of HER2 + BC

**Fig. 1:**
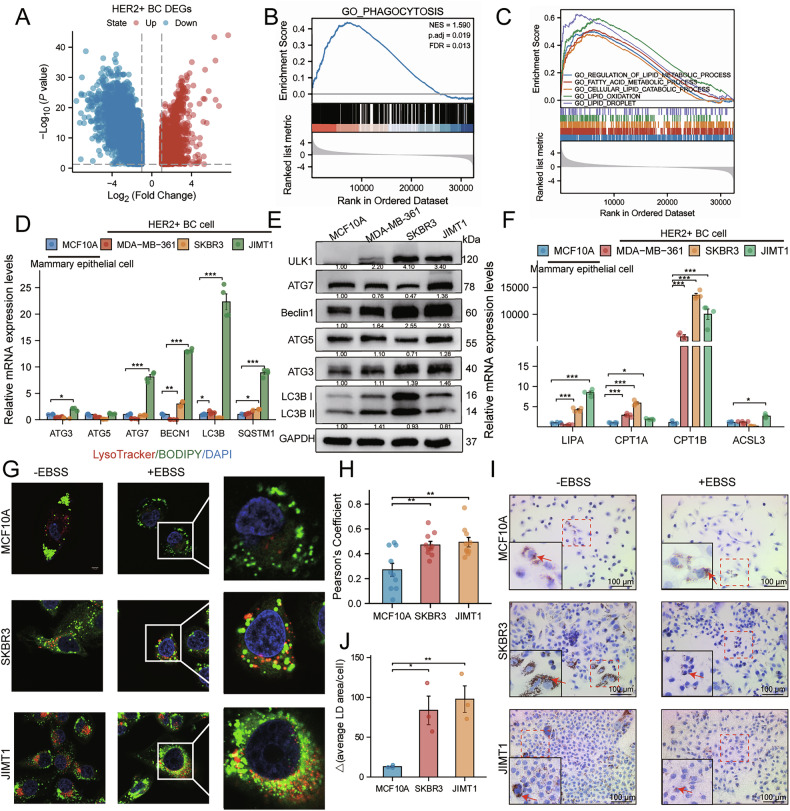
Enhanced lipophagy flux is a metabolic signature of HER2 + BC. **A** Volcano plot depicting differentially expressed genes (DEGs) in HER2 + BC (*n* = 180) vs normal tissues (*n* = 22) from the TCGA-BRCA dataset. Upregulated genes are in red, downregulated in blue (fold change > 2.0, adjusted *p* < 0.05). **B**, **C** GSEA showing enrichment of phagocytosis-related (**B**) and lipid metabolism (**C**) pathways in HER2 + BC. **D**, **E** RT-qPCR (**D**; *n* = 4) and western blot (**E**) analysis of core autophagy markers in HER2 + BC cell lines vs the non-tumorigenic mammary epithelial cell line MCF10A. **F** RT-qPCR analysis (*n* = 4) of key lipid catabolism genes across the indicated cell lines. **G**, **H** Representative images (**G**) and quantification (**H**, *n* = 10) of lysosome (LysoTracker Red) and LD (BODIPY) co-localization. **I**, **J** Representative images (**I**) and quantification (**J**) of LDs (Oil Red O staining) in cells cultured in standard medium (-EBSS) or starved in EBSS for 4 h. The change in LD area (Δ) reflects starvation-induced clearance. Data are mean ± SEM. ^*^p < 0.05, ^**^p < 0.01, ^***^p < 0.001.

To define the molecular features of HER2 + BC, we analyzed transcriptomic data from 180 HER2 + BC and 22 paired normal samples from the TCGA-BRCA database. Differential gene expression analysis (fold change > 2.0, adjusted *p* < 0.05) revealed a distinct transcriptional profile in HER2 + BC (Fig. 1A). GSEA revealed a significant upregulation of phagocytosis-related gene signatures (Fig. 1B) and, notably, an enrichment of pathways associated with lipid catabolism and oxidation (Fig. 1C). Given that lipophagy is a selective autophagic process central to lipid degradation [16], we hypothesized that lipophagic activity is elevated in HER2 + BC.

Consistent with this hypothesis, RT-qPCR and WB assays demonstrated significantly higher expression of core autophagy markers, such as Beclin1 (BECN1) and microtubule-associated protein 1 A/1B-light chain 3B (LC3B), in HER2 + BC cells compared to the non-tumorigenic mammary epithelial cells MCF10A (Fig. 1D–E and Supplementary Fig. S1A). Furthermore, the mRNA expression of *LIPA* [32] and other key enzymes in lipid degradation and β-oxidation (*CPT1A* [33], *CPT1B* [34], and *ACSL3* [35]) was markedly upregulated in HER2 + BC cells (Fig. 1F). Fluorescence co-localization assays confirmed increased interaction between lysosomes and LDs in HER2 + BC cells vs controls, indicative of heightened lipophagic flux (Fig. 1G, H). Consequently, starvation-induced LD clearance was accelerated in HER2 + BC cells, signifying rapid LD catabolism (Fig. 1I, J).

To ascertain the HER2-dependency of this phenotype, we examined the effects of HER2 inhibition with trastuzumab. In trastuzumab-sensitive SKBR3 cells, LC3B-II/I ratios exhibited a biphasic response, while lipid catabolic genes were sustainably upregulated. In contrast, trastuzumab-resistant JIMT1 cells showed a progressive increase in LC3B-II/I ratios but only a transient induction of lipid catabolic genes (Supplementary Fig. S2A–B). These cell-type-specific patterns suggest that HER2 signaling dynamically coordinates lipophagic responses to metabolic stress.

### TM9SF1 is a prognostic biomarker associated with lipophagy in HER2 + BC

**Fig. 2:**
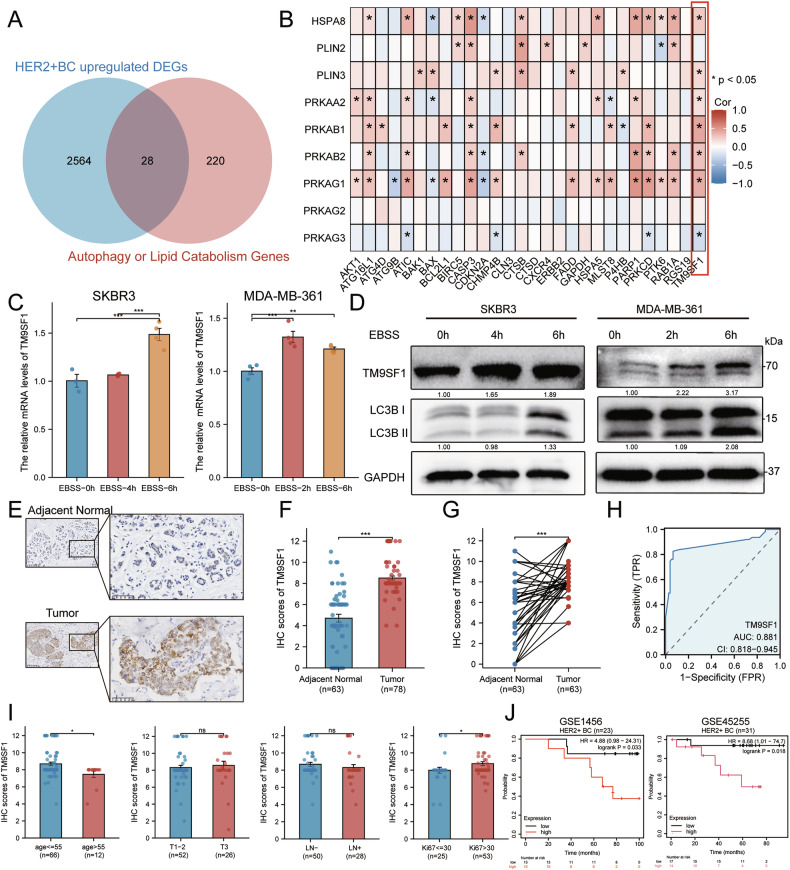
TM9SF1 is a clinically relevant prognostic biomarker associated with lipophagy in HER2 + BC. **A** Venn diagram showing the intersection of upregulated genes (fold change > 1.333, adjusted *p* < 0.002) in HER2 + BC with curated autophagy and lipid catabolism gene sets to identify 28 candidate regulators. **B** Heatmap of Spearman’s correlation between the 28 candidate genes and nine known lipophagy effectors. **C**, **D** RT-qPCR (**C**; *n* = 4) and western blot (**D**) analysis of TM9SF1 expression in cells starved for the indicated times. **E** Representative immunohistochemical images of TM9SF1 in HER2 + BC tissues and paired adjacent normal breast tissues. **F**, **G** Quantification of TM9SF1 expression from immunohistochemical staining in unpaired (**F**; adjacent normal = 63, tumor = 78) and paired (**G**; *n* = 63) patient cohorts. **H** Receiver operating characteristic curve analysis of TM9SF1 as a diagnostic biomarker for HER2 + BC, AUC area under the curve. **I** Association between TM9SF1 expression and clinicopathological features. **J** Kaplan–Meier analysis of overall survival in two independent HER2 + BC cohorts, stratified by *TM9SF1* expression. Left: GSE1456 cohort (*n* = 23). Right: GSE45255 cohort (*n* = 31). Data are mean ± SEM. ^*^p < 0.05, ^**^p < 0.01, ^***^p < 0.001.

Currently, research on lipophagy-related regulators in HER2 + BC is still in its early stages. To identify novel genes potentially regulating lipophagy in HER2 + BC, we collected 230 autophagy-related genes from the Human Autophagy Database and 29 lipid catabolism-associated genes from the Molecular Signatures Database, yielding a total of 248 unique genes. These genes intersected with 2,592 highly differentially expressed genes (fold change > 1.333, adjusted *p* < 0.002) in HER2 + BC derived from the TCGA-BRCA dataset, resulting in 28 candidate genes (Fig. 2A). Spearman’s correlation analysis between these candidates and known lipophagy-related genes revealed that *TM9SF1* exhibited a relatively strong positive correlation with key lipophagy markers (Fig. 2B). Among the TM9SF members, TM9SF1 showed the strongest association with core lipophagy effectors (e.g., *PRKAB1*, *PRKAG1*, and *BECN1*), whereas *TM9SF4* showed minimal pathway connectivity (Supplementary Fig. S3A). Experimentally, we confirmed that under starvation conditions, TM9SF1 expression was significantly upregulated, in parallel with an increase in the LC3B-II/I ratio (Fig. 2C, D and Supplementary Fig. S1B).

To validate these findings clinically, immunohistochemistry analysis of our patient cohort confirmed that TM9SF1 expression in HER2 + BC tissues was significantly higher than that in adjacent normal tissues (tumor = 78 vs normal = 63) (Fig. 2E–G). Receiver operating characteristic curve analysis demonstrated that TM9SF1 is a strong diagnostic biomarker for HER2 + BC, with an area under the curve of 0.881 (Fig. 2H). High TM9SF1 expression was also associated with high-risk clinical features, including younger age and high Ki67 indices (Fig. 2I). To explore the prognostic relevance of TM9SF1, we analyzed its association with overall survival in two independent publicly available datasets. In both the GSE1456 (*n* = 23) and GSE45255 (*n* = 31) cohorts, Kaplan-Meier analysis revealed a consistent trend where high *TM9SF1* expression was associated with poorer patient outcomes (Fig. 2J). Consistent with its role as a stress-responsive mediator, TM9SF1 expression showed biphasic dynamics following HER2 inhibition (Supplementary Fig. S3B). Taken together, these data identify TM9SF1 as a clinically relevant, lipophagy-associated prognostic biomarker in HER2 + BC.

### TM9SF1 promotes HER2 + BC cell growth and proliferation in vitro and in vivo

**Fig. 3:**
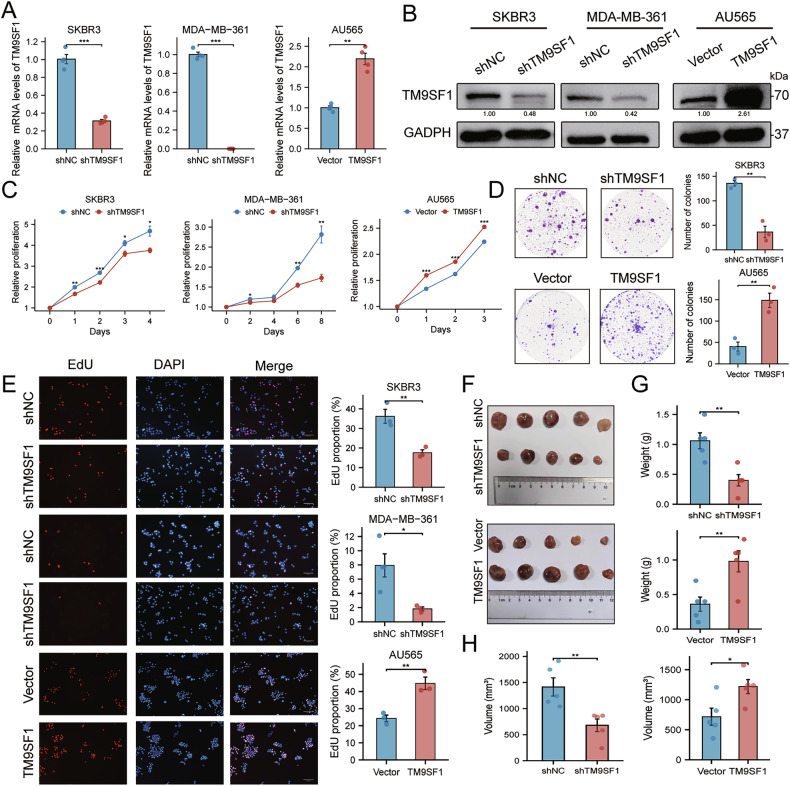
TM9SF1 promotes HER2 + BC growth and proliferation in vitro and in vivo. **A**, **B** Validation of TM9SF1 knockdown (shTM9SF1) and overexpression (TM9SF1) efficiency by RT-qPCR (**A**; *n* = 4) and western blot (**B**). **C**–**E** Proliferation of control, shTM9SF1, and TM9SF1-overexpression cells assessed by CCK-8 (based on OD450 nm values) (**C**), colony formation (**D**), and EdU incorporation (**E**) assays. **F**–**H** In vivo tumor growth of xenografts derived from control, shTM9SF1, or TM9SF1-overexpression cells (*n* = 5 per group). Representative tumor images (**F**), tumor weights (**G**), and tumor volume (**H**) at endpoint are shown. Data are mean ± SEM. ^*^p < 0.05, ^**^p < 0.01, ^***^p < 0.001.

To determine TM9SF1’s role in HER2 + BC, we generated stable cell lines with either knockdown or overexpression of TM9SF1, validated at both the mRNA and protein levels (Fig. 3A, B). A battery of proliferation assays, including CCK8 (Fig. 3C), colony formation (Fig. 3D), and EdU incorporation (Fig. 3E), consistently demonstrated that TM9SF1 knockdown significantly inhibited the growth of HER2 + BC cells. Conversely, TM9SF1 overexpression promoted cell proliferation. These in vitro findings were recapitulated in vivo, where tumors derived from TM9SF1-overexpressing cells grew significantly larger and faster than control tumors, while tumors from TM9SF1-knockdown cells showed stunted growth (Fig. 3F–H).

### TM9SF1 is essential for autophagic flux and LD turnover

**Fig. 4:**
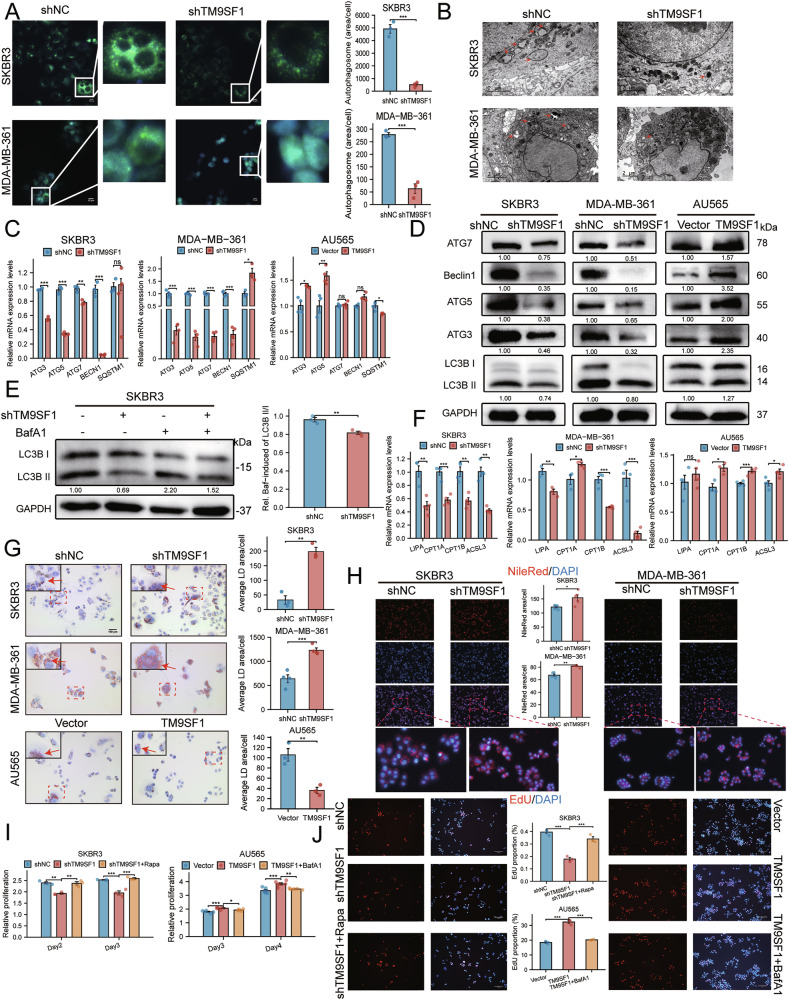
TM9SF1 is essential for autophagic flux and LD turnover. **A** Representative images and quantification of autophagosomes visualized by MDC staining. **B** Representative TEM images showing autophagosomes (red arrows). **C**, **D** RT-qPCR (**C**; *n* = 4) and western blot (**D**) analysis of autophagy-related markers in cells with altered TM9SF1 expression. **E** Western blot analysis of LC3B-II/I levels in control and shTM9SF1 cells treated with or without BafA1 (100 nM, 1 h) to assess autophagic flux. **F** RT-qPCR analysis of key acidic lipolytic genes. **G**, **H** Representative images and quantification of LDs by Oil Red O (**G**) and Nile Red (**H**; *n* = 4) staining. **I**, **J** CCK8 (based on OD450 nm values) (**I**) /EdU (**J**) assays showing that rapamycin (10 nM, 24 h) rescues the growth defect of shTM9SF1 cells, while BafA1 (100 nM, 1 h) abrogates the growth advantage of TM9SF1-overexpression cells. Data are mean ± SEM. ^*^p < 0.05, ^**^p < 0.01, ^***^p < 0.001.

To dissect the mechanism underlying TM9SF1-driven proliferation, we first investigated its role in autophagy, a process implicated by our initial bioinformatic screen. Both MDC staining (Fig. 4A) and TEM (Fig. 4B) revealed a significant reduction in autophagosomes upon TM9SF1 depletion. Spearman’s correlation analysis revealed that the expression levels of *TM9SF1* and autophagy-related genes were positively correlated, with a particularly significant positive correlation with the expression of *BECN1* (*ρ* = 0.543, *p* < 0.001) (Supplementary Fig. S3A). RT-qPCR (Fig. 4C) and WB (Fig. 4D and Supplementary Fig. S1C) revealed that TM9SF1 knockdown decreased, whereas its overexpression increased, the expression of autophagic markers, including lipidated LC3B-II. An autophagic flux assay using the lysosomal inhibitor bafilomycin A1 (BafA1) [36] confirmed that TM9SF1 knockdown impairs autophagosome biogenesis rather than accelerating autolysosome clearance (Fig. 4E).

Concurrently, TM9SF1 knockdown suppressed the expression of key lipolytic genes, including *LIPA*, *CPT1A*, *CPT1B*, and *ACSL3* (Fig. 4F). This transcriptional suppression manifested phenotypically as a significant accumulation of LDs, as observed by Oil Red O (Fig. 4G) and Nile Red staining (Fig. 4H). Conversely, TM9SF1 overexpression promoted LD clearance. Importantly, the anti-proliferative effect of TM9SF1 knockdown was rescued by the autophagy inducer rapamycin, while the pro-proliferative effect of TM9SF1 overexpression was abrogated by BafA1 (Fig. 4I–J). Collectively, these results demonstrate that TM9SF1 enhances autophagic flux and promotes the lysosomal degradation of LDs, while also facilitating HER2 + BC cell proliferation through enhanced autophagy.

### TM9SF1 regulates lipophagy via the AMPK-ULK1 signaling axis

**Fig. 5:**
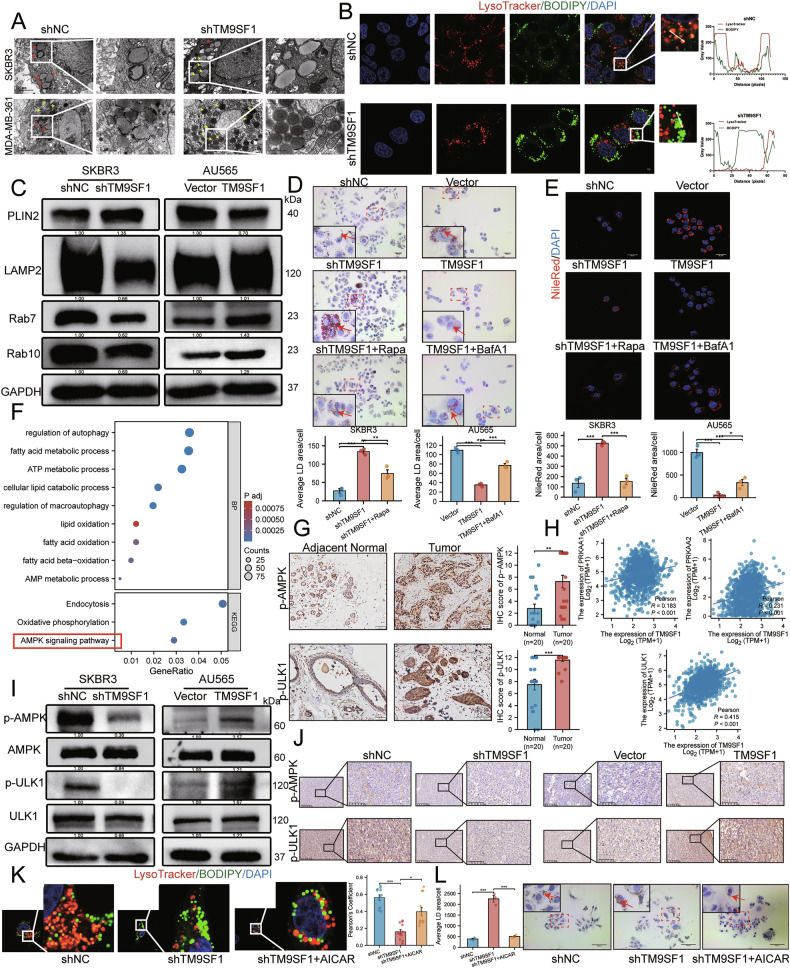
TM9SF1 regulates lipophagy via the AMPK-ULK1 signaling axis. **A** Representative TEM image showing lipophagosomes (red arrows) and LDs (yellow arrows). **B** Representative images and quantification of lysosome-LD co-localization. **C** Western blot analysis of lipophagy-related proteins. LAMP2 lysosome-associated membrane protein 2, PLIN2 perilipin 2 (LD surface protein). **D**, **E** Pharmacological modulation of LD turnover. Rapamycin (10 nM, 24 h) reverses LD accumulation in shTM9SF1 cells, while BafA1 (100 nM, 1 h) restores LDs in TM9SF1-overexpression cells. **F** KEGG pathway enrichment analysis of differentially expressed proteins from proteomics data comparing shTM9SF1 and control SKBR3 cells. **G** Representative immunohistochemical images of p-AMPK and p-ULK1 in HER2 + BC vs adjacent normal tissues (*n* = 20). **H** Correlation analysis between *TM9SF1* and *PRKAA1*/*PRKAA2*/*ULK1* expression in the TCGA-BRCA cohort. **I** Western blot analysis of AMPK (p-Thr172) and ULK1 (p-Ser555) phosphorylation. **J** Representative immunohistochemical images of p-AMPK and p-ULK1 in xenograft tumors. **K**, **L** The AMPK activator AICAR (1 mM, 1 h) rescues the defects in lysosome-LD co-localization (K; *n* = 12) and reverses LD accumulation (L) in shTM9SF1 cells. Data are mean ± SEM. ^*^p < 0.05, ^**^p < 0.01, ^***^p < 0.001.

To precisely visualize the impact of TM9SF1 on lipophagy, we used TEM to examine LD-containing autophagic vesicles. In control cells, double-membraned lipophagosomes were readily observed encapsulating LDs. In contrast, these structures were markedly reduced in shTM9SF1 cells, which instead accumulated cytosolic LDs (Fig. 5A). This observation was corroborated by LysoTracker/BODIPY fluorescence microscopy, which showed a significant decrease in lysosome-LD co-localization in shTM9SF1 cells (Fig. 5B). The WB results further revealed that TM9SF1 expression positively correlated with lipophagy flux markers (lysosome-associated membrane protein 2 [LAMP2], RAS oncogene family members RAB7 [37] and RAB10 [38]), while negatively correlating with the LD-coating protein perilipin 2 (PLIN2) (Fig. 5C, Supplementary Fig. S1D). To further confirm whether the degradation of LDs is regulated by TM9SF1-mediated lipophagy, we used rapamycin [39] and BafA1 to modulate LD numbers in response to changes in TM9SF1 expression. Oil Red O (Fig. 5D) and Nile Red staining (Fig. 5E) showed that rapamycin reversed LD accumulation in TM9SF1-knockdown cells, whereas BafA1 restored the reduction in LDs caused by TM9SF1 overexpression. These findings provide further evidence supporting the critical role of TM9SF1 in promoting lipophagy flux.

To elucidate the underlying mechanism by which TM9SF1 regulates lipophagy, we performed quantitative proteomics on SKBR3-shTM9SF1 cells. Functional enrichment analysis identified a significant enrichment of differential expression proteins in the AMPK signaling pathway (Fig. 5F). The AMPK/ULK1 signaling pathway plays a critical role in autophagy as a key upstream regulator. Based on these findings, we hypothesized that TM9SF1 may influence lipophagy levels through the AMPK/ULK1 pathway. Immunohistochemical analysis of clinical samples revealed significantly higher levels of phosphorylated AMPK (p-AMPK) and ULK1 (p-ULK1) in HER2 + BC tissues compared to normal tissues (*n* = 20) (Fig. 5G). TCGA-BRCA transcriptomic data further showed a positive correlation between *TM9SF1* and the expression of AMPK subunits (*PRKAA1*, *PRKAA2*) and *ULK1* expression (Fig. 5H). Crucially, WB analysis confirmed this regulatory link: knockdown of TM9SF1 attenuated the phosphorylation of AMPK at Thr172 and consequently suppressed the phosphorylation of its downstream target ULK1 at Ser555 (Fig. 5I and Supplementary Fig. S1E). This regulatory cascade was recapitulated in tumor xenografts (Fig. 5J). Treatment with the AMPK activator AICAR [40] rescued the lipophagic defect and reversed LD accumulation in shTM9SF1 cells (Fig. 5K, L). Although co-immunoprecipitation assays did not detect a direct interaction between TM9SF1 and AMPK (Supplementary Fig. S4A), these findings strongly suggest that TM9SF1 sustains lipophagy by maintaining the activity of the AMPK-ULK1 signaling axis.

### TM9SF1-driven lipophagy fuels fatty acid release and energy production

**Fig. 6:**
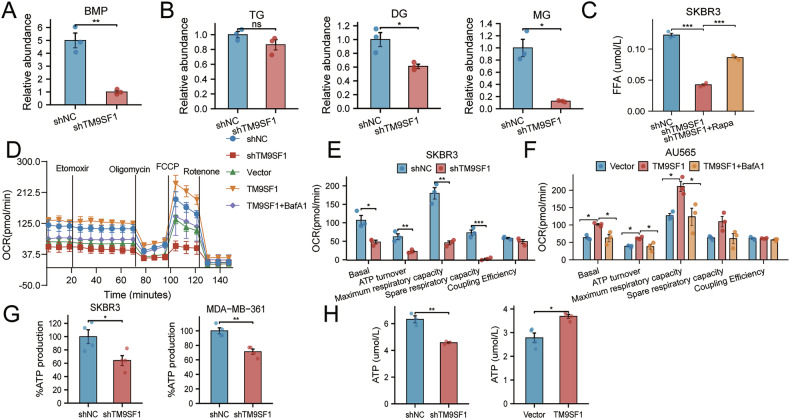
TM9SF1-driven lipophagy fuels fatty acid release and energy production. **A**, **B** Relative abundance of Bis(monoacylglycerol)phosphate (BMP; **A**) and other lipid species (triglycerides [TG], diglycerides [DG], and monoglycerides [MG]; **B** in control vs shTM9SF1 cells, as determined by lipidomics. **C** Intracellular FFA levels. Rapamycin (10 nM, 24 h) rescues the FFA deficit in shTM9SF1 cells. **D**–**F** Seahorse analysis of oxygen consumption rate (OCR) showing the contribution of FAO. OCR curve (**D**), OCR in shTM9SF1 cells (**E**), and OCR in TM9SF1-overexpression cells treated with or without BafA1 (100 nM, 1 h) (**F**). **G** Cellular ATP levels in cultured cells (**G**; *n* = 4) and xenograft tumors (**H**). Data are mean ± SEM. ^*^p < 0.05, ^**^p < 0.01, ^***^p < 0.001.

To delineate the metabolic consequences of TM9SF1-mediated lipophagy, we performed liquid chromatography-tandem mass spectrometry-based lipidomics. Bis(monoacylglycerol)phosphate, a lysosomal phospholipid essential for lipophagic LD turnover [41, 42], was significantly reduced upon TM9SF1 knockdown (Fig. 6A). Concomitant decreases in monoglycerides and diglycerides (Fig. 6B) further indicated impaired LD catabolism, as these intermediates arose from sequential decomposition processing of triglycerides. Critically, TM9SF1 depletion reduced intracellular FFAs, which were the terminal products of lipophagy, and this deficit was rescued by rapamycin (Fig. 6C), directly linking TM9SF1 to functional lipophagic LD turnover.

As lipophagy is critical for maintaining cellular energy balance, we next assessed its bioenergetic impact. A Seahorse metabolic flux analysis revealed that TM9SF1 promotes fatty acid β-oxidation to generate energy flux, a process that was inhibited by BafA1 (Fig. 6D–F). Consistent with this, ATP assays demonstrated that TM9SF1 knockdown led to a significant decrease in ATP production in both cell types (Fig. 6G) and in vivo mouse tumor tissues (Fig. 6H), confirming that TM9SF1-driven lipophagy is essential for energy generation.

### TM9SF1 enhances metabolic fitness and survival under nutrient stress

**Fig. 7:**
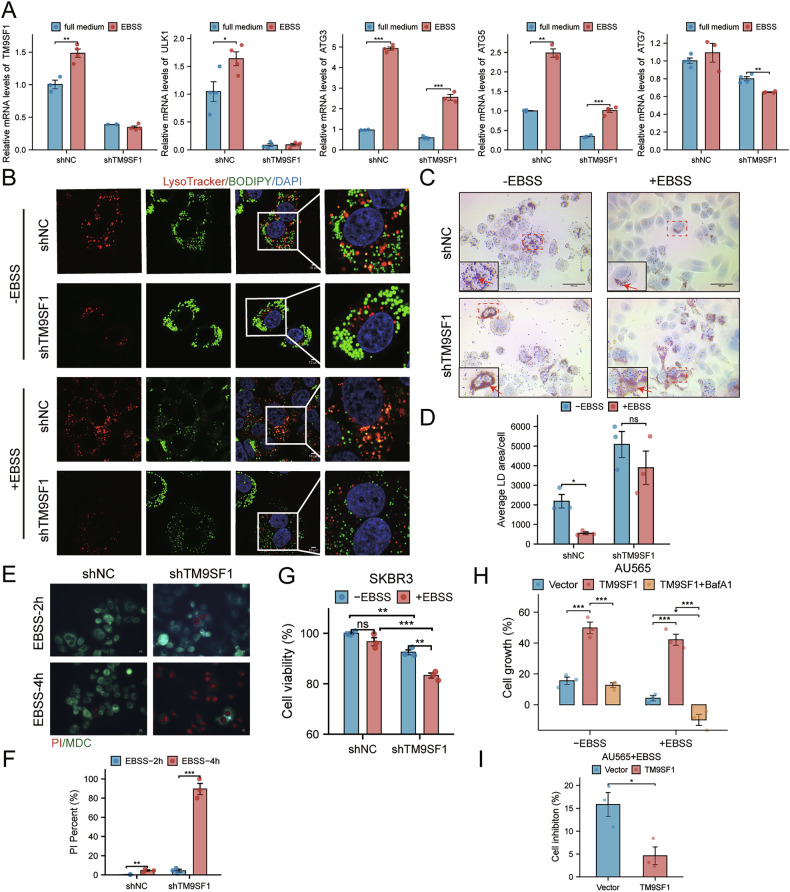
TM9SF1 enhances metabolic fitness and survival under nutrient stress. **A** RT-qPCR analysis of autophagy markers in control and shTM9SF1 cells cultured in standard medium or starved in EBSS for 6 h (*n* = 4). **B** Representative images of lysosome-LD co-localization in cells starved for 4 h. **C**, **D** Representative images and quantification of LDs in cells starved for 4 h. **E**, **F** Representative images (**E**) and quantification (**F**) of cell death by Propidium Iodide (PI) staining after 2 or 4 h of starvation. **G**–**I** Proliferation of shTM9SF1 (**G**) and TM9SF1-overexpression (**H**, **I**) cells under nutrient stress (EBSS, 2 h), assessed by CCK8. BafA1 (100 nM, 1 h) was used to inhibit lipophagy. Data are mean ± SEM. ^*^p < 0.05, ^**^p < 0.01, ^***^p < 0.001.

**Fig. 8:**
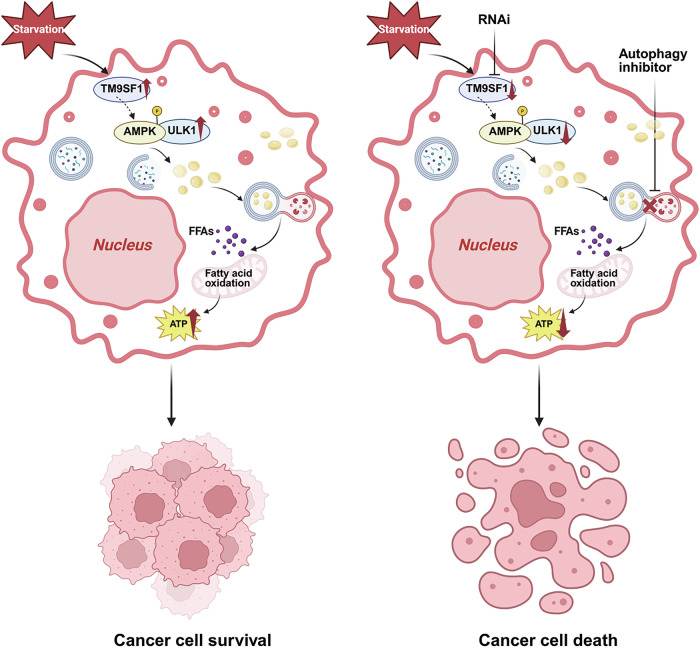
Schematic model of TM9SF1-mediated regulation of lipophagy in HER2 + BC. TM9SF1 maintains the activity of the AMPK-ULK1 signaling axis to drive lipophagic flux. This process facilitates the breakdown of LDs into FFAs, which fuel mitochondrial β-oxidation to sustain cellular energetics. This metabolic adaptation enhances the survival and proliferation of HER2 + BC cells, particularly under nutrient stress. Figure created with BioRender.com.

Lipophagy enables cancer cells to maintain energy homeostasis and survive nutrient deprivation by mobilizing LDs for β-oxidation. To test this, we subjected cells to starvation using EBSS. Starvation stress robustly induced autophagic markers in control cells, but this response was blunted in shTM9SF1 cells (Fig. 7A). Consequently, while control cells increased lipophagy flux under starvation, shTM9SF1 cells failed to enhance lysosome-LD co-localization (Fig. 7B) or accelerate LD clearance (Fig. 7C, D). As a result, shTM9SF1 cells exhibited significantly reduced viability and proliferation under starvation compared to control cells (Fig. 7E–G). Conversely, TM9SF1-overexpressing cells showed enhanced growth capacity and resistance to starvation-induced growth inhibition. This survival advantage was abrogated by BafA1, indicating its dependency on lipophagy (Fig. 7H, I). Collectively, these findings establish that TM9SF1 enhances the metabolic fitness of HER2 + BC cells during nutrient deprivation by sustaining lipophagic flux. The overall proposed mechanism is summarized in Fig. 8.

## Discussion

Our study establishes TM9SF1 as a pivotal regulator of metabolic reprogramming in HER2 + BC, orchestrating a lipophagy-dependent mechanism that sustains tumor cell survival and proliferation under metabolic stress. We demonstrate that TM9SF1 is upregulated in HER2 + BC, where it correlates with poor prognosis. Mechanistically, TM9SF1 drives lipophagic flux by maintaining the activation of the AMPK-ULK1 signaling cascade, thereby facilitating the degradation of LDs to fuel mitochondrial β-oxidation and maintain energy homeostasis. These findings uncover a critical vulnerability in HER2 + BC, presenting a compelling rationale for targeting this metabolic axis to overcome therapeutic resistance.

Metabolic reprogramming, particularly the dysregulation of lipid metabolism, is a hallmark of aggressive malignancies [43, 44], including HER2 + BC. These tumors exhibit heightened dependence on LD catabolism and fatty acid β-oxidation to sustain rapid proliferation and resist metabolic stress [7, 45, 46]. By demonstrating for the first time that TM9SF1 amplifies lipophagy to shuttle LDs to lysosomes for degradation into FFAs, we provide a mechanistic link between a specific membrane protein and the bioenergetic fortitude of cancer cells. This finding not only expands upon previous reports linking FAO to HER2 + BC progression [47, 48] but also positions TM9SF1 as an upstream gatekeeper of this entire process, ensuring metabolic and lipophagic integrity.

Central to this regulatory network is the AMPK-ULK1 axis, which TM9SF1 engages to promote lipophagy. Although AMPK-ULK1 signaling is a well-established mediator of canonical autophagy, our work delineates its specific role in TM9SF1-driven lipophagy. The marked reduction in AMPK and ULK1 phosphorylation upon TM9SF1 knockdown, and the subsequent reversal of lipophagic defects by an AMPK activator, firmly positions TM9SF1 as a critical upstream modulator of this pathway. The absence of direct interaction with AMPK suggests the involvement of intermediate factors. We propose two plausible, non-mutually exclusive hypotheses: (1) TM9SF1 may recruit lipid kinases, such as phosphatidylinositol 4-kinase IIIβ (PI4KIIIβ) [49], to facilitate AMPK activation at specific membrane microdomains, analogous to liver kinase B1 (LKB1)-AMPK regulation at the lysosome [50]; or (2) TM9SF1 could function as a scaffold to stabilize membrane receptor complexes essential for LD turnover, as observed in other lipophagy contexts [27]. Future studies are warranted to dissect these possibilities.

From a clinical standpoint, the upregulation of TM9SF1 in HER2 + BC and its association with poor prognosis are significant. This aligns with the adverse outcomes linked to other lipid metabolism genes, such as fatty acid synthase (*FASN*) and cluster of differentiation 36 (*CD36*), in this subtype [45, 51], suggesting that a dependency on lipophagy may be a shared vulnerability of aggressive HER2+ tumors. While the patient numbers in the analyzed Gene Expression Omnibus (GEO) cohorts were limited, the consistent trend observed across two independent datasets provides preliminary evidence for the prognostic value of TM9SF1 in HER2 + BC. Future studies in larger, well-annotated prospective cohorts are warranted to validate these findings and establish TM9SF1 as a robust clinical biomarker. This TM9SF1-lipophagy network may represent a core mechanism of intrinsic and acquired resistance to targeted therapies. Indeed, our preliminary data suggest that HER2 signaling itself dynamically coordinates autophagic and lipolytic programs in response to trastuzumab, with distinct adaptive patterns emerging in sensitive vs resistant cells. This implies that targeting TM9SF1 could disrupt a fundamental survival strategy employed by tumors under therapeutic pressure.

These findings have profound therapeutic implications. While autophagy inhibitors (e.g., chloroquine) and FAO inhibitors (e.g., etomoxir) have shown promise in preclinical models [52, 53], their broad mechanisms may lead to off-target effects. Our work suggests that targeting the TM9SF1-lipophagy axis could offer a more specific strategy to dismantle the metabolic resilience of advanced tumors. A multi-pronged approach, co-targeting oncogenic HER2 signaling and this specific metabolic dependency, could overcome the heterogeneity and adaptability that currently limit the efficacy of HER2-targeted therapies. Looking forward, several important questions remain. Delineating the precise interplay between HER2 signaling and TM9SF1-mediated lipophagy, and exploring its connection with anabolic pathways like sterol regulatory element-binding protein 1 (SREBP1)-driven lipogenesis [54], will be critical. Furthermore, exploring whether TM9SF1 expression could serve as a predictive biomarker for response to metabolic therapies warrants investigation in future clinical trials.

In conclusion, our study delineates a TM9SF1-AMPK-ULK1 regulatory axis that sustains lipid metabolic fitness in HER2 + BC by driving lipophagic flux. This work illuminates a novel mechanism of metabolic adaptation and provides a robust rationale for targeting TM9SF1-mediated lipophagy as a strategy to disrupt the metabolic scaffolds that support tumor aggressiveness and therapeutic resistance.

## Supplementary information

Supplementary Information

## Data Availability

Data from the TCGA-BRCA database is available to the public (https://portal.gdc.cancer.gov/). The mass spectrometry proteomics data reported in this paper have been deposited in the Figshare repository under the 10.6084/m9.figshare.30073645.
