# Supplementary material for: TM9SF1 drives the lipophagic flux via AMPK-ULK1 signaling to sustain metabolic fitness in HER2-positive breast cancer
Source: Cell Death Dis. 2025 Oct 24;16(1):755. doi: 10.1038/s41419-025-08093-y (PMC12552452; doi:10.1038/s41419-025-08093-y)

**Supplementary Information for**TM9SF1 Drives the Lipophagic Flux via AMPK-ULK1 Signaling to Sustain Metabolic Fitness in HER2-Positive Breast Cancer

Xiaofen Li^1*^, Xiaoqin Yu^2*^, Kaiyan Huang^3*^, Xin Yu^1*^, Shiping Luo^1^, Xiewei Huang^1^, Chuangui Song^1^

^1^Department of Breast Surgery, Clinical Oncology School of Fujian Medical University, Fujian Cancer Hospital (Fujian Branch of Fudan University Shanghai Cancer Center), Fuzhou, China.

^2^Fujian Medical University, Fuzhou, China.

^3^Department of Breast Surgery, The Second Affiliated Hospital of Fujian Medical University, Quanzhou 362000, China.

**Correspondence:**

Chuangui Song: songcg1971@outlook.com; ORCID: 0009-0008-0749-568X

**This file includes:**

Figs. S1 to S4 and legends

Table S1 to S3

Unedited western blot images

**Supplementary Figures**

**
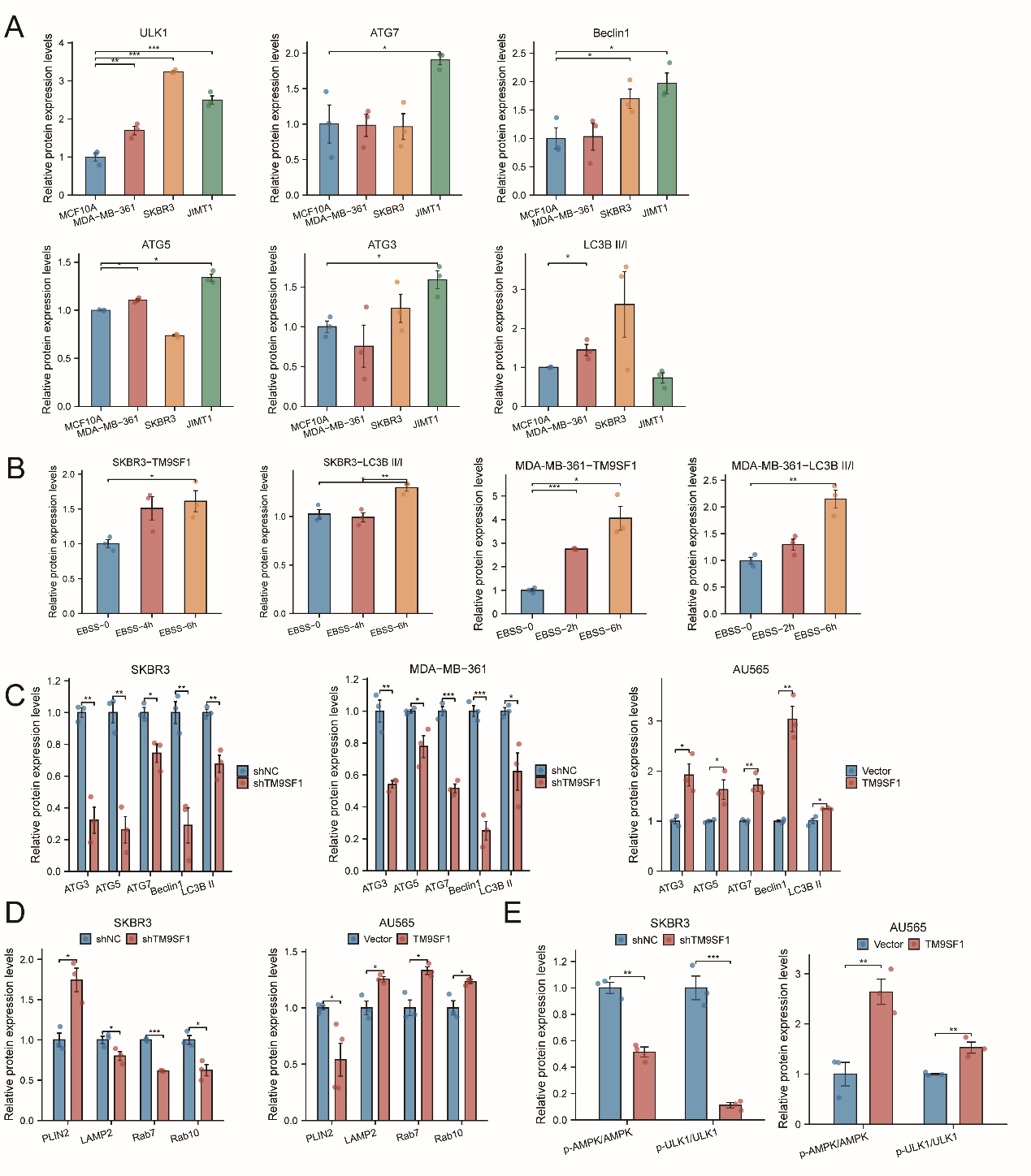
**

**Supplementary Fig. S1.** **Quantification of western blot data.** Quantification of protein levels from three independent experiments for blots shown in (A) Fig. 1E, (B) Fig. 2D, (C) Fig. 4D, (D) Fig. 5C, and (E) Fig. 5I. Data were normalized to GAPDH. Data are mean ± SEM. **p* < 0.05, ***p* < 0.01, ****p* < 0.001.


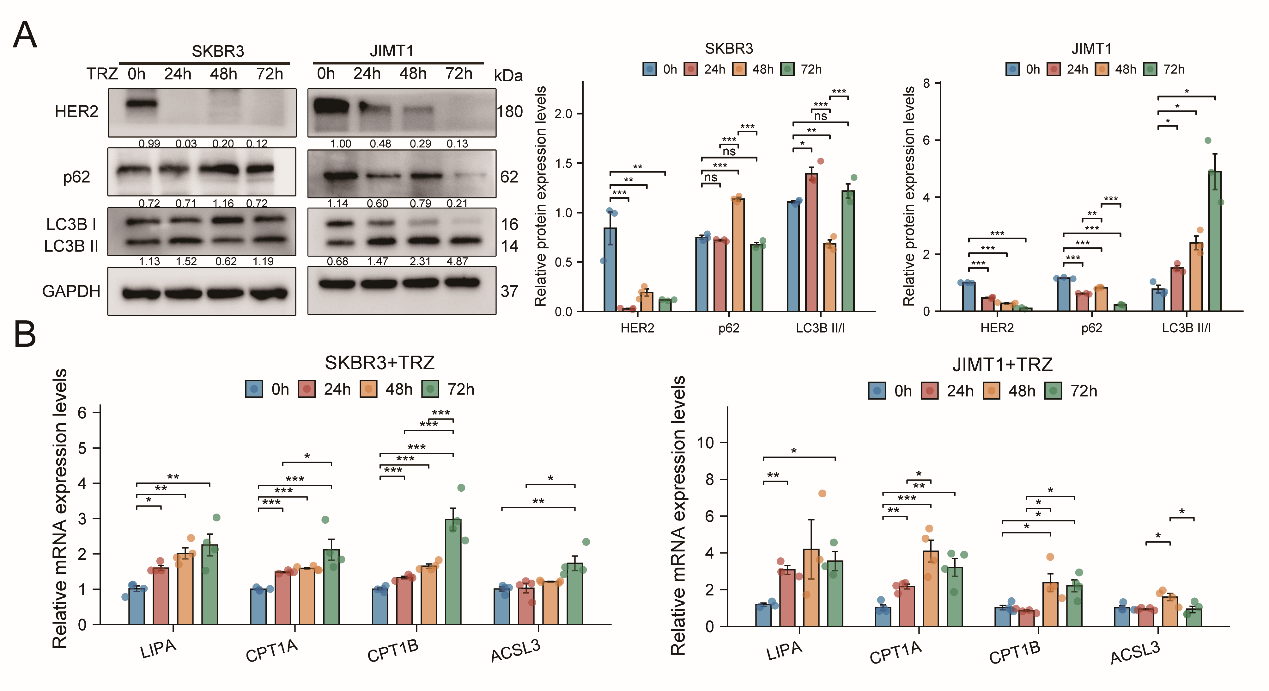


**Supplementary Figure S2. Trastuzumab dynamically coordinates autophagic flux and lipid catabolism.** (A) Western blotting analysis of p62 and LC3B-II/I ratio in trastuzumab-sensitive SKBR3 (1 mg/mL) and resistant JIMT1 (2 mg/mL) cells treated for the indicated durations. (B) RT-qPCR analysis (n = 4) of lipid catabolism-related transcripts in cells treated as in (A). Data are mean ± SEM. **p* < 0.05, ***p* < 0.01, ****p* < 0.001.


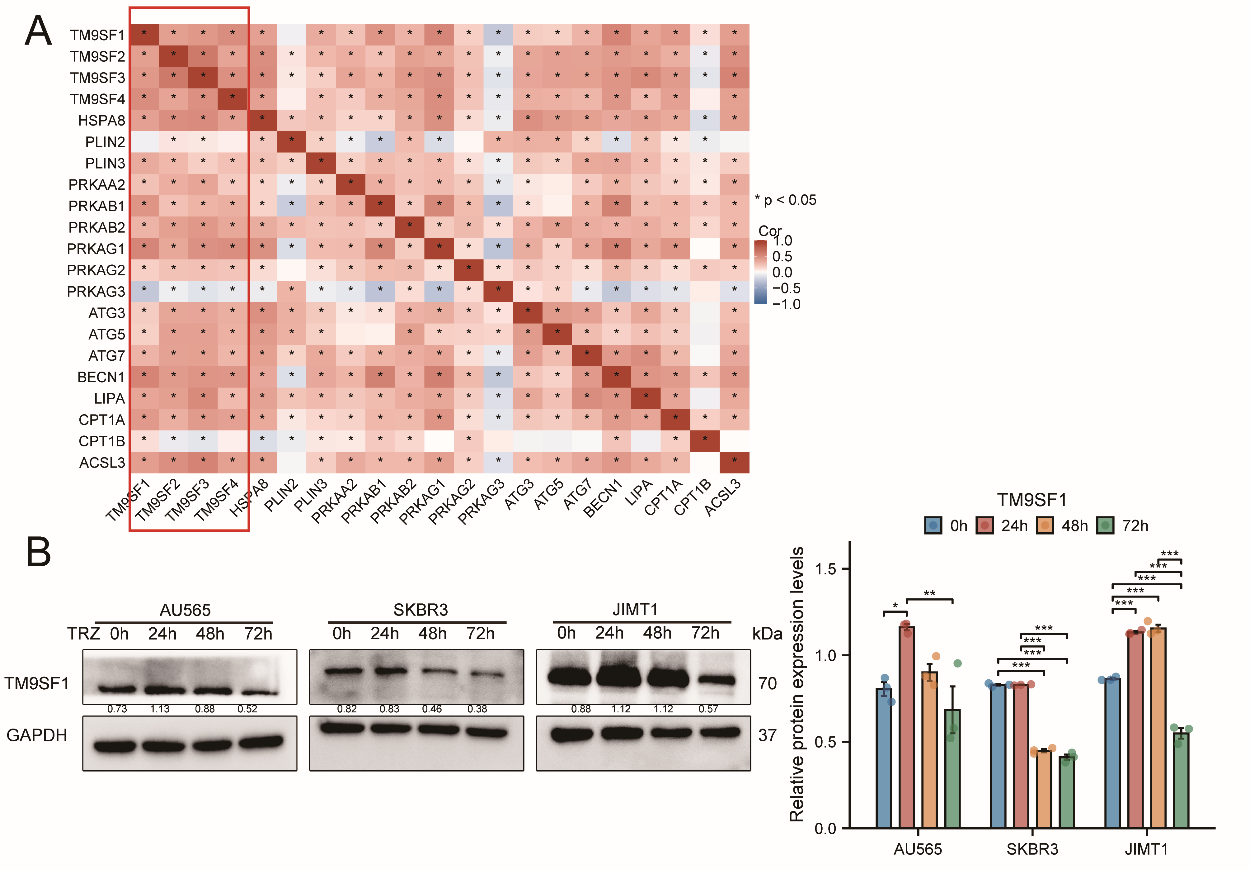


**Supplementary Fig. S3. TM9SF1 expression correlates with lipophagy genes and is modulated by HER2 inhibition.** (A) Heatmap of Spearman’s correlation between TM9SF members and core lipophagy-related genes. (B) Western blot analysis of TM9SF1 expression in HER2+ BC cell lines treated with trastuzumab for the indicated durations. Data are mean ± SEM. **p* < 0.05, ***p* < 0.01, ****p* < 0.001.


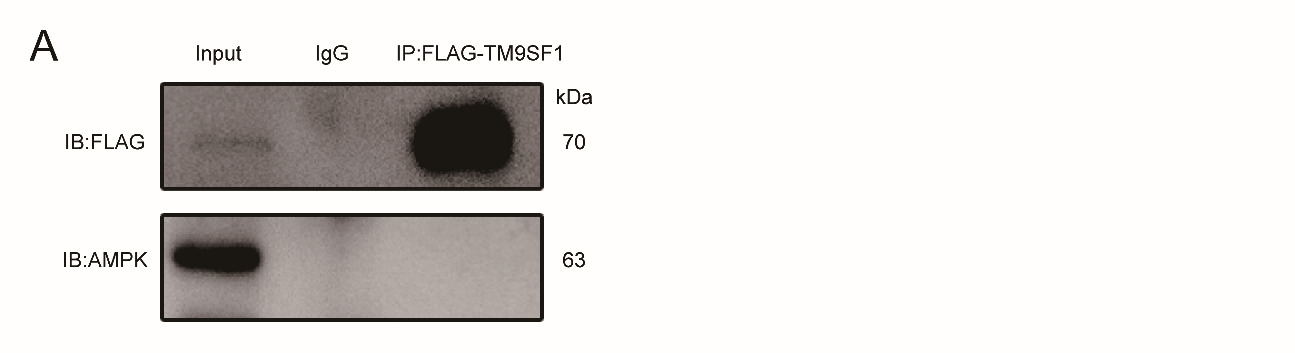


**Supplementary Fig. S4.** **TM9SF1 does not directly interact with AMPK.** (A) Co-immunoprecipitation assays using antibodies against flag or AMPK in HEK293T cells. Input lanes confirm protein expression, while IP lanes show no co-precipitation.

**Supplementary Tables**

**Table S1. The sequences of shRNAs used in this study.**

| NO. | Target Sequence |
| --- | --- |
| shTM9SF1 | GCACTGGCATTATTGTCATGG |
| shNC | TTCTCCGAACGTGTCACGT |

**Table S2. Gene-specific primers used for qRT-PCR.**

| Gene |  | Sequence (5’→3’) |
| --- | --- | --- |
| TM9SF1 | FORWARD | CTGCATCTCTGGCTACGTGT |
| TM9SF1 | REVERSE | AGTTCACCACACTCCACGTC |
| ATG3 | FORWARD | CTGGCGGTGAAGATGCTATT |
| ATG3 | REVERSE | GCTGCCGTTGCTCATCATAG |
| ATG5 | FORWARD | AGAAGCTGTTTCGTCCTGTGG |
| ATG5 | REVERSE | AGGTGTTTCCAACATTGGCTC |
| ATG7 | FORWARD | GGTGTGAATGCCAGAGGATT |
| ATG7 | REVERSE | CCATCAATAGGAAGACGACATCAT |
| BECN1 | FORWARD | GGTGTCTCTCGCAGATTCATC |
| BECN1 | REVERSE | TCAGTCTTCGGCTGAGGTTCT |
| SQSTM1 | FORWARD | TCGGATAACTGTTCAGGAGGAG |
| SQSTM1 | REVERSE | TCGGATTCTGGCATCTGTAGG |
| MAP1LC3B | FORWARD | \| ACCCTGAGTCTTCTCTTCAGG \| \| --- \| \|  \| |
| MAP1LC3B | REVERSE | AGTTTACAGTCAGGGCCGTT |
| ULK1 | FORWARD | TTCCAAACACCTCGGTCCTC |
| ULK1 | REVERSE | GCTCAGGGATGGTTCCAACT |
| LIPA | FORWARD | TGTGGGTCATTCTCAAGGCA |
| LIPA | REVERSE | GCTAGTACAGAAGGCGACGG |
| CPT1A | FORWARD | GGAGATTATCAACAAGCCAGACC |
| CPT1A | REVERSE | CACACCATAGCCGTCATCAG |
| CPT1B | FORWARD | GTAAGTTCTGCCTGACCTATGAG |
| CPT1B | REVERSE | CGGTACATATTCTGGTGCTTCTT |
| ACSL3 | FORWARD | AGTGCTTTCCGAAGCTGCTAT |
| ACSL3 | REVERSE | CCAGACCAGTTTCAGGGGTC |
| GAPDH | FORWARD | GGTGTGAACCATGAGAAGTATGA |
| GAPDH | REVERSE | GAGTCCTTCCACGATACCAAAG |

**Table S3. Information on the primary and secondary antibodies used in the study.**

| Antibody | Suppliers | Cat# | Host | Working concentration | Molecular Weight (kDa) |
| --- | --- | --- | --- | --- | --- |
| TM9SF1 | Invitrogen | PA5-84406 | Rabbit | 1:100(IHC) | / |
| TM9SF1 | Immunoway | YN2876 | Rabbit | 1:1000(WB) | 66 |
| ATG3 | Proteintech | 11262-2-AP | Rabbit | 1:1000(WB) | 40 |
| ATG5 | Proteintech | 10181-2-AP | Rabbit | 1:1000(WB) | 55 |
| ATG7 | Proteintech | 81760-1-RR | Rabbit | 1:1000(WB) | 78 |
| Beclin1 | Proteintech | 11306-1-AP | Rabbit | 1:1000(WB) | 60 |
| p62 | Zenbio | 380612 | Rabbit | 1:1000(WB) | 62 |
| LC3B | Abcam | ab51520 | Rabbit | 1:1000(WB) | 14/16 |
| PLIN2 | Immunoway | YN2883 | Rabbit | 1:1000(WB) | 48 |
| LAMP2 | Zenbio | R23340 | Rabbit | 1:1000(WB) | 120 |
| Rab7 | Proteintech | 55469-1-AP | Rabbit | 1:1000(WB) | 23 |
| Rab10 | Proteintech | 11808-1-AP | Rabbit | 1:1000(WB) | 23 |
| p-ULK1 (Ser757) | Cell Signaling | 6888 | Rabbit | 1:1000(WB) | 140 |
| ULK1 | Zenbio | R381887 | Rabbit | 1:1000(WB)  1:100 (IHC) | 120 |
| p-AMPK (Thr172) | Cell Signaling | 2535 | Rabbit | 1:1000(WB)  1:100 (IHC) | 62 |
| AMPK | Cell Signaling | 2532 | Rabbit | 1:1000 (WB) | 62 |
| DYKDDDDK tag antibody (Binds to FLAG® tag epitope) | Proteintech | 66008-4-Ig | Mouse | 0.5-4.0 ug for 1.0-3.0 mg of total protein lysate (IP) | / |
| Anti-DDDDK tag | Abcam | ab205606 | Rabbit | 1:3000 (WB) | / |
| GAPDH | Proteintech | 60004-1-Ig | Mouse | 1:7500 (WB) | 37 |
| HRP-Goat Anti-Rabbit Recombinant Secondary Antibody | Proteintech | RGAR001 | Goat | 1:10000 (WB) | / |
| HRP-Goat Anti-Mouse Recombinant Secondary Antibody | Proteintech | RGAM001 | Goat | 1:10000 (WB) | / |

**Unedited western blot images**


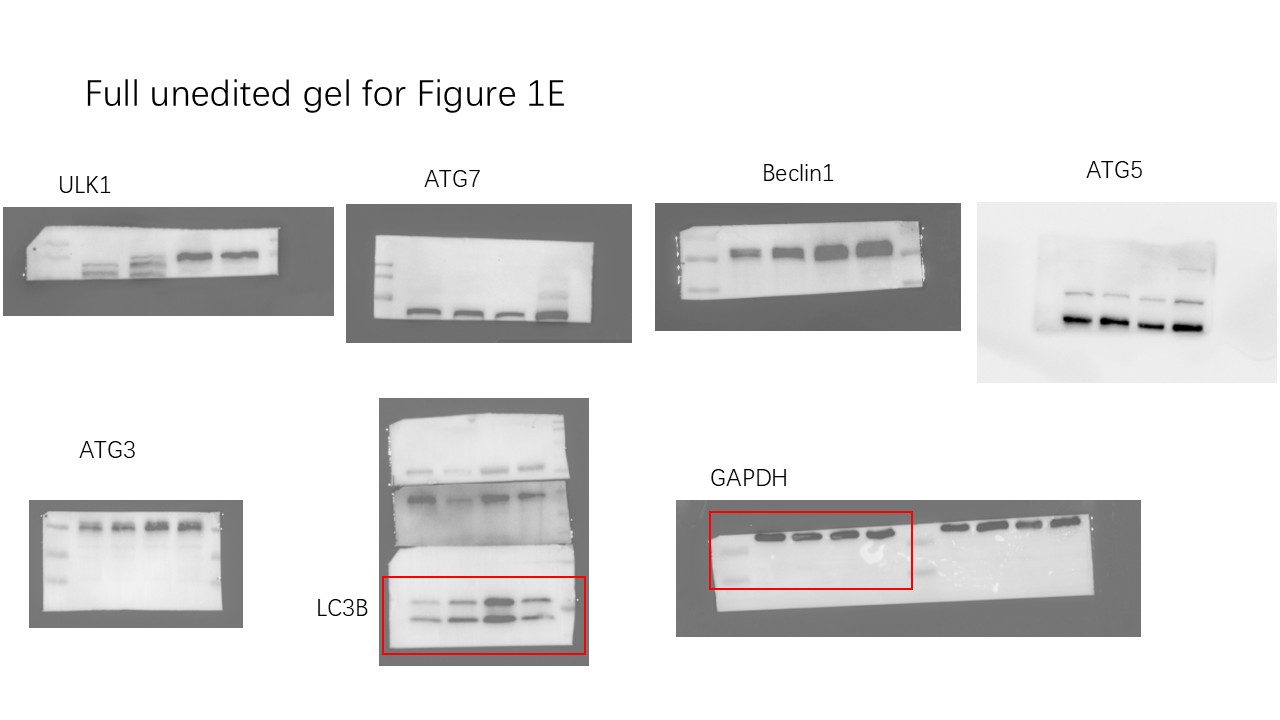


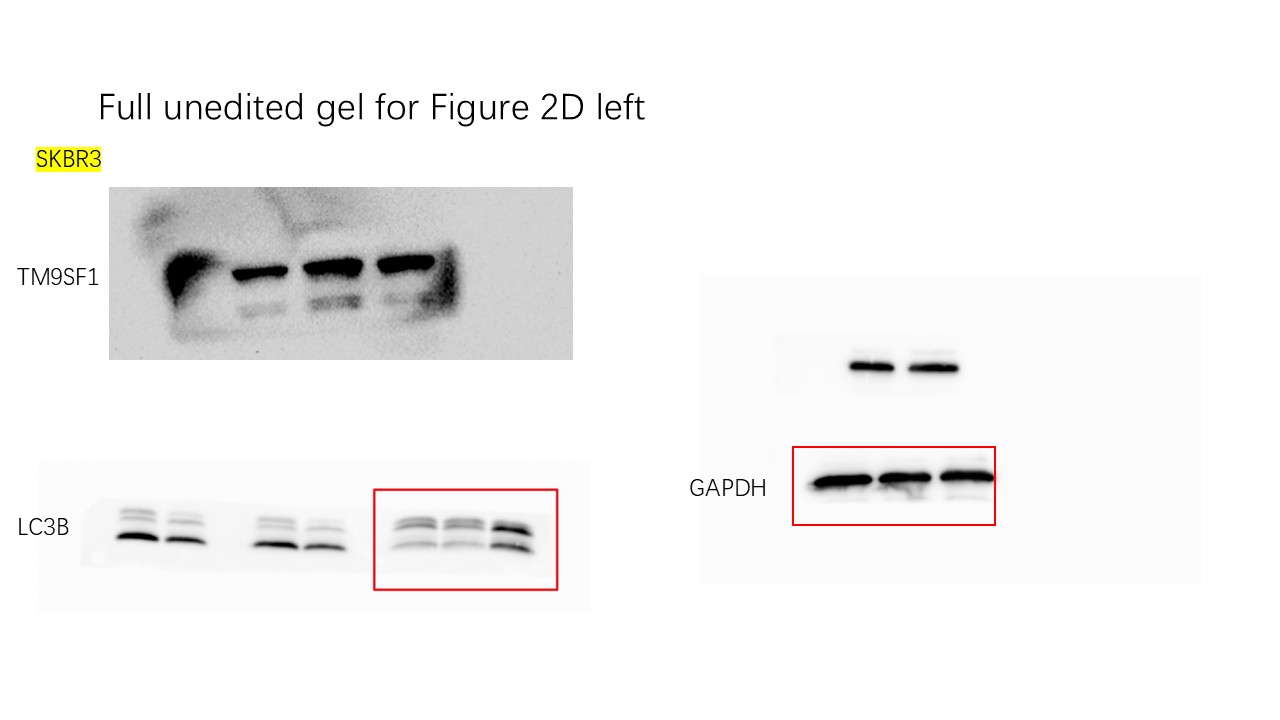


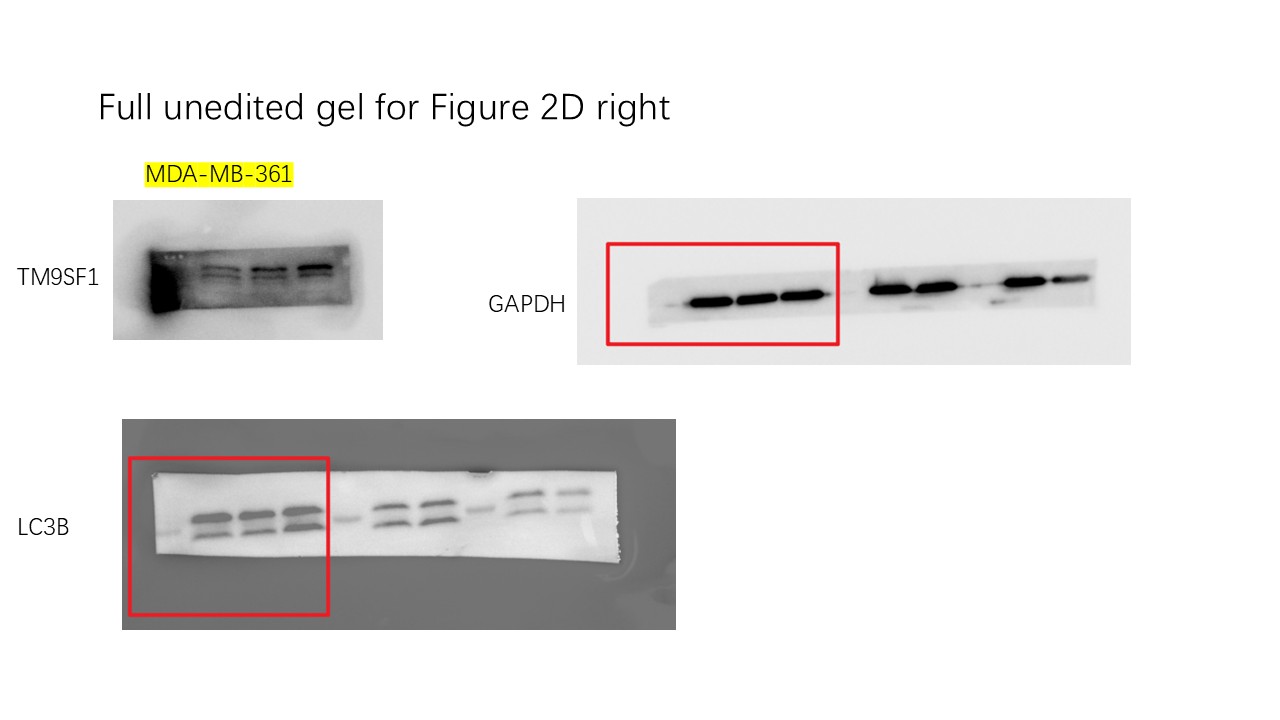


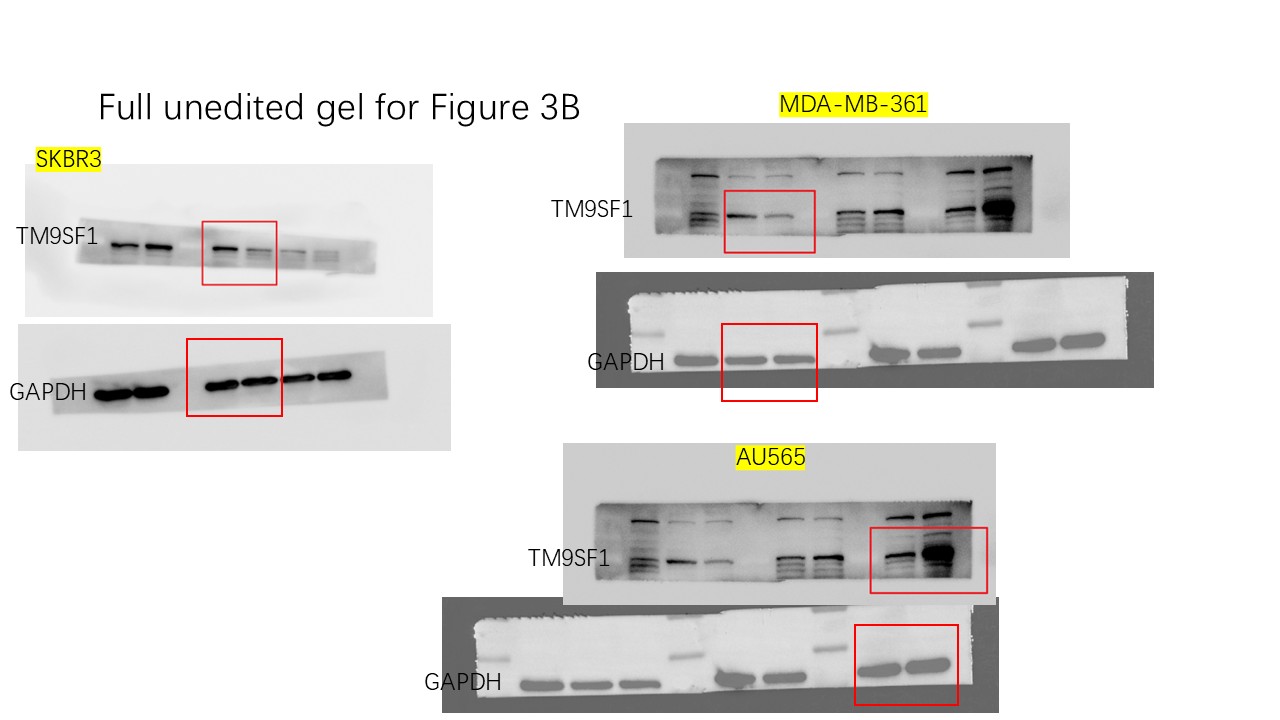


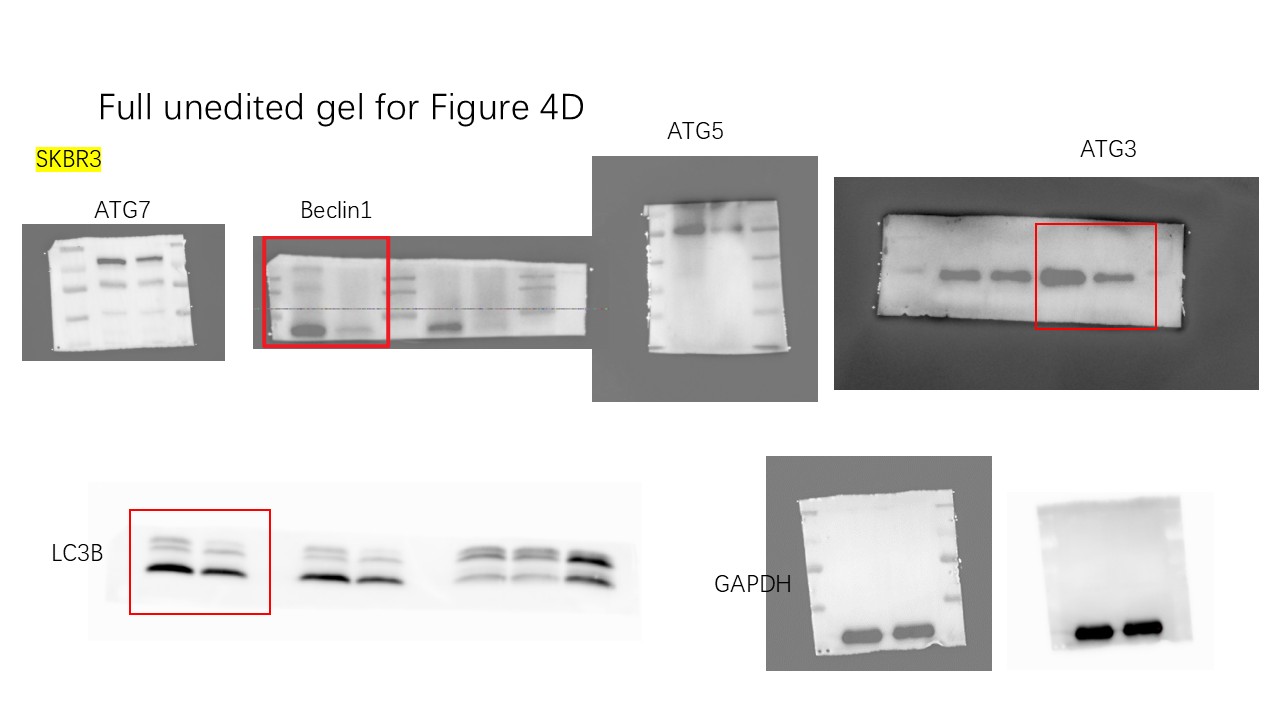


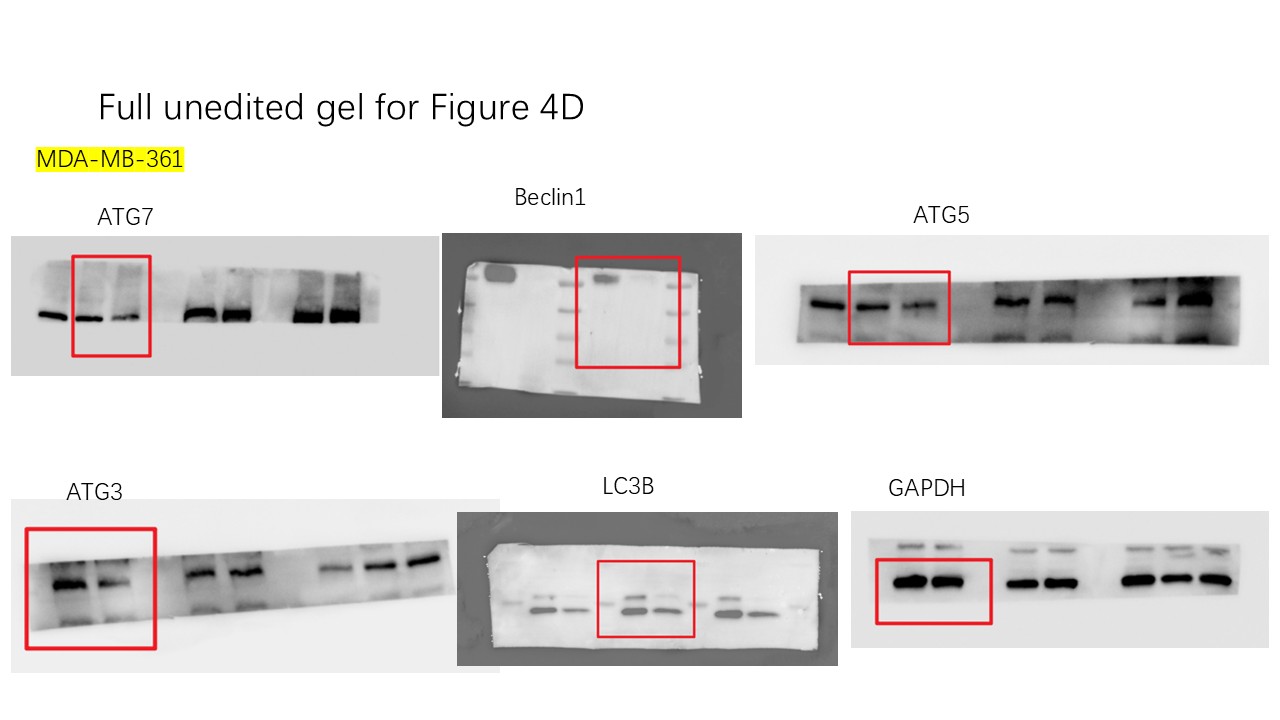


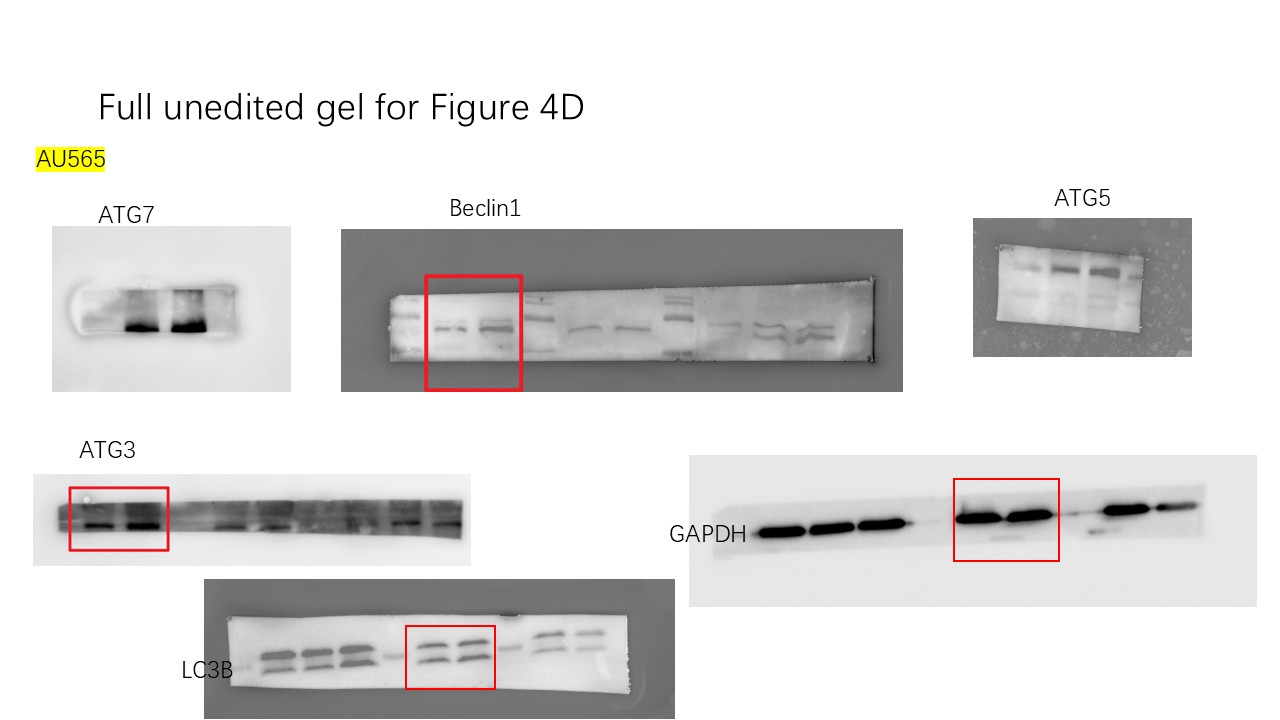


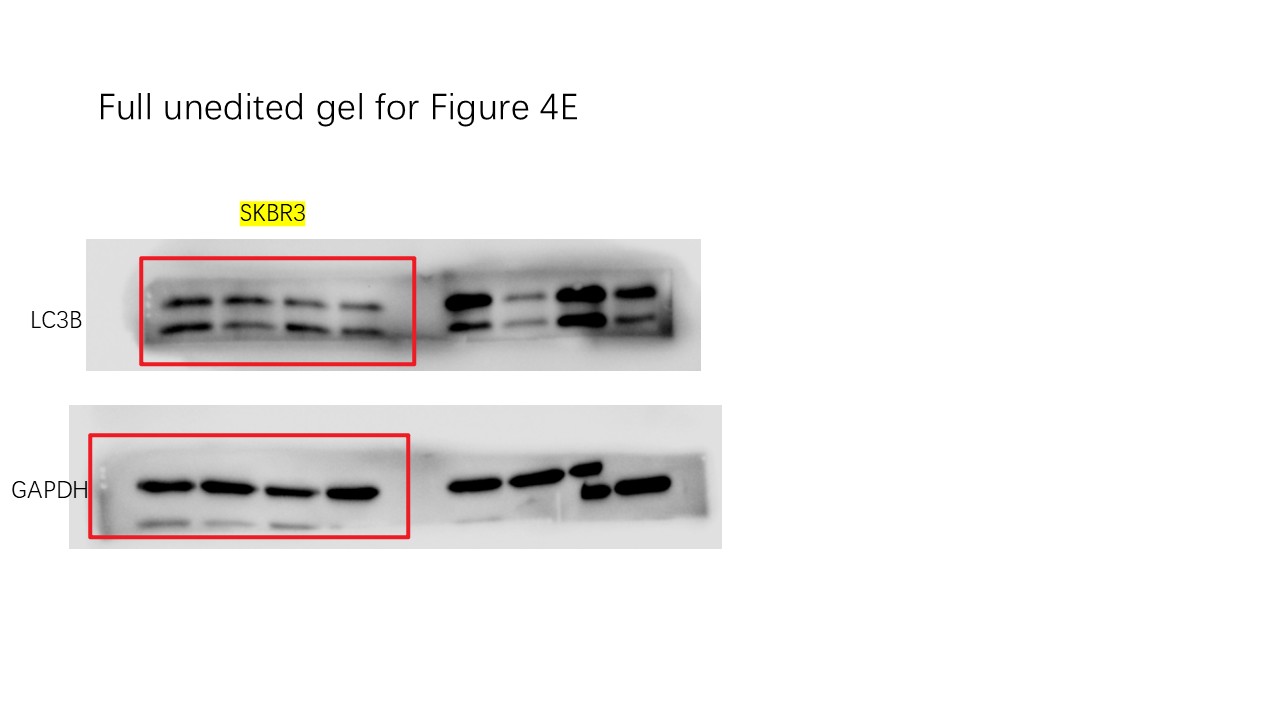


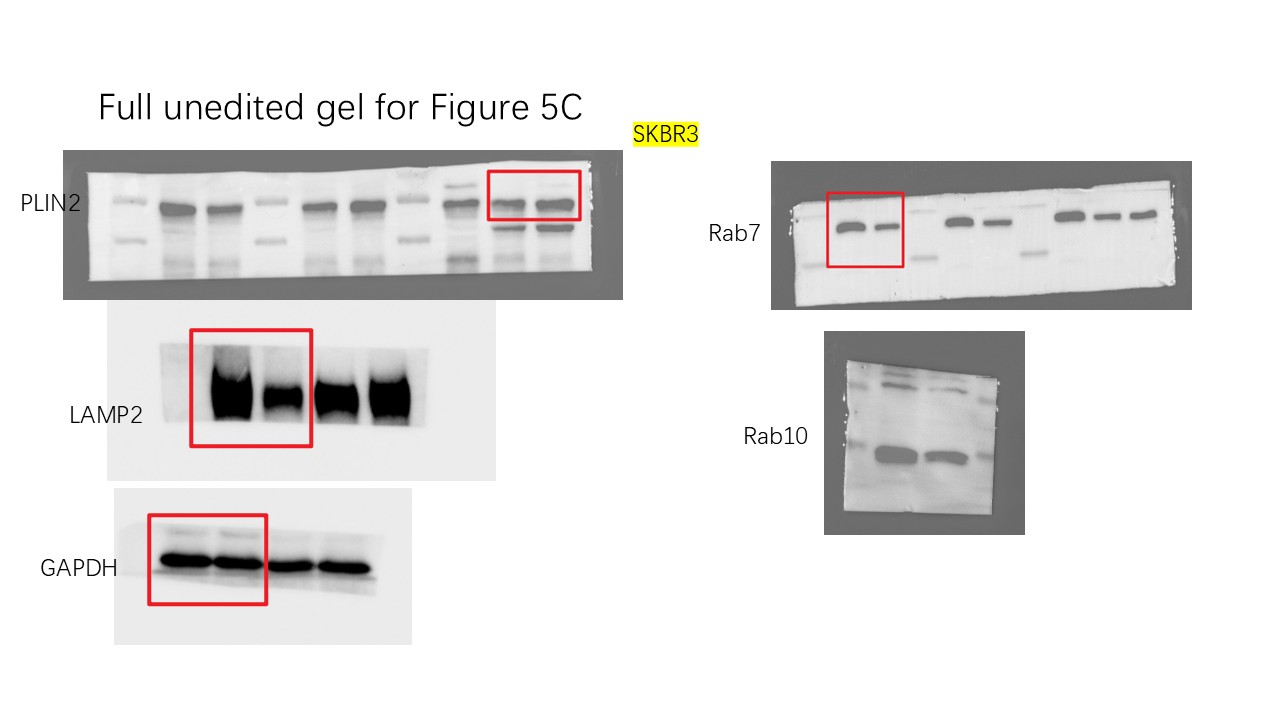


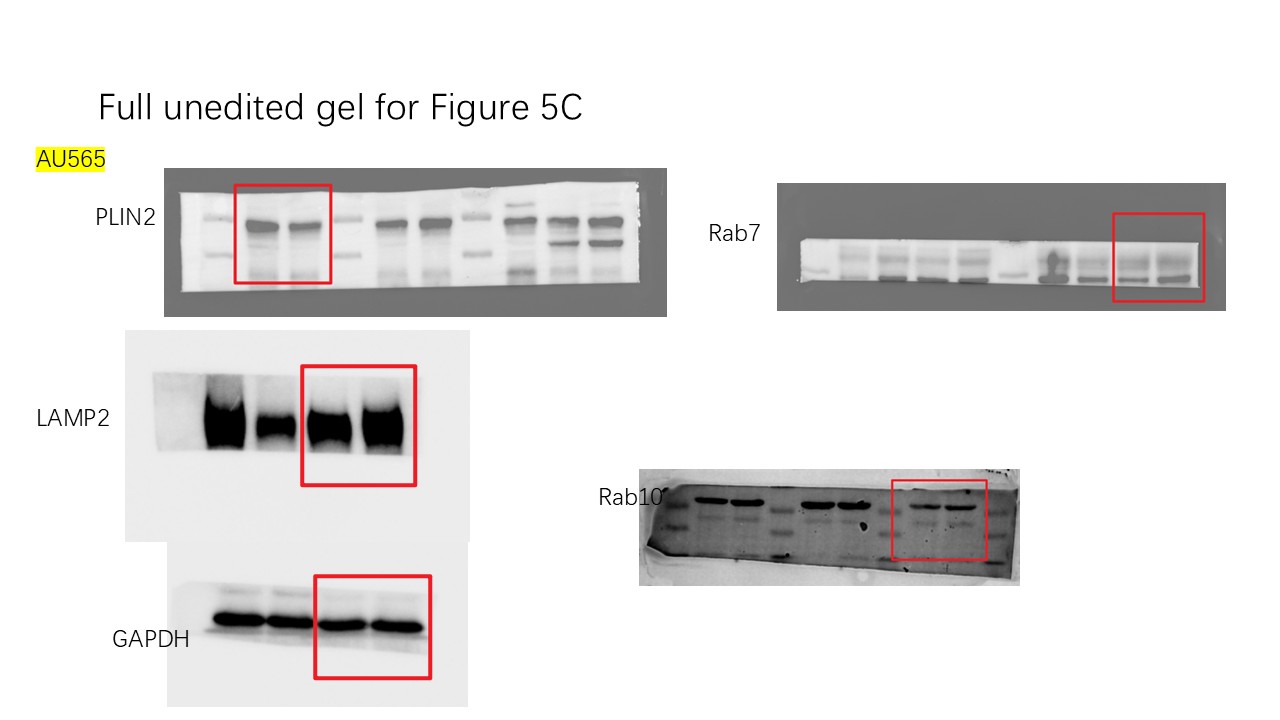


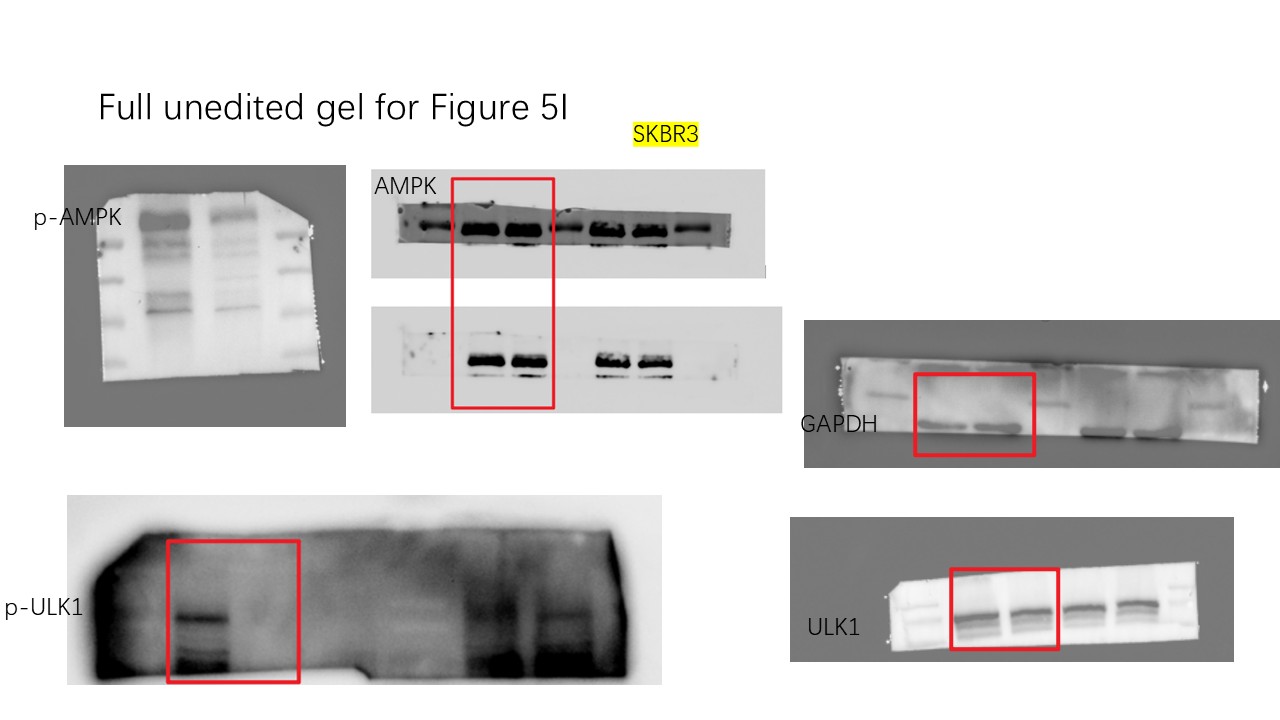


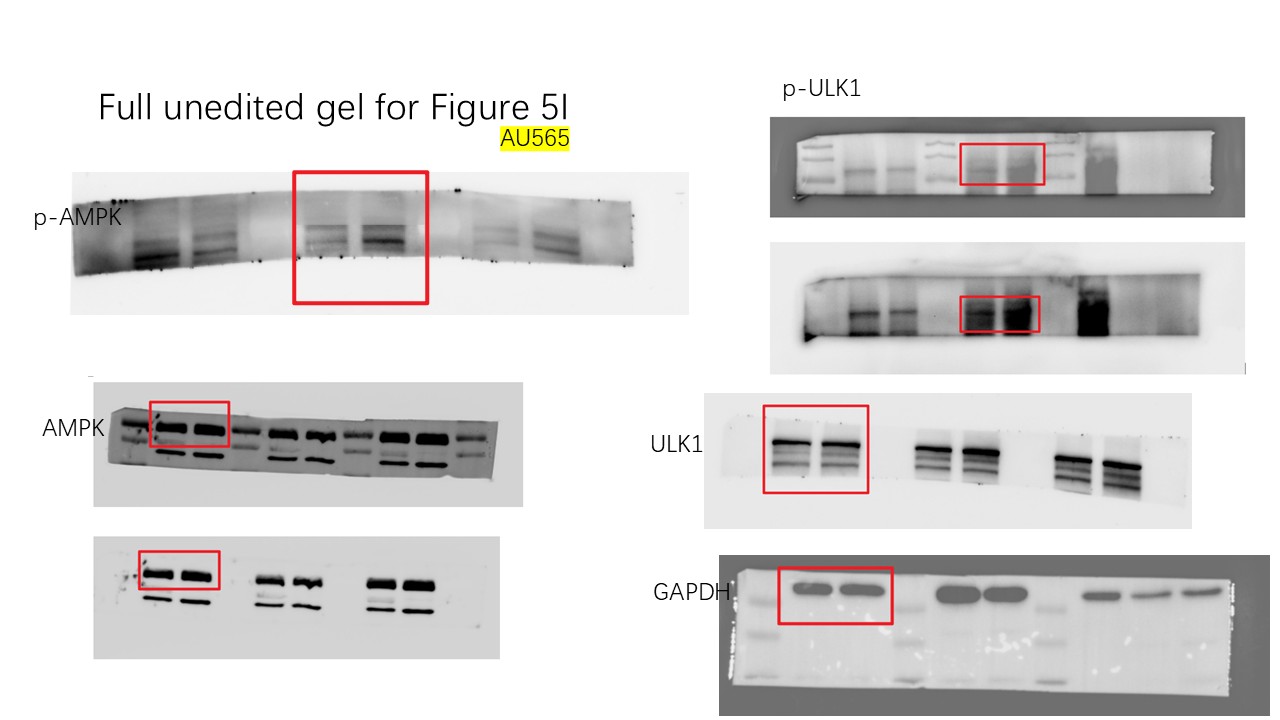


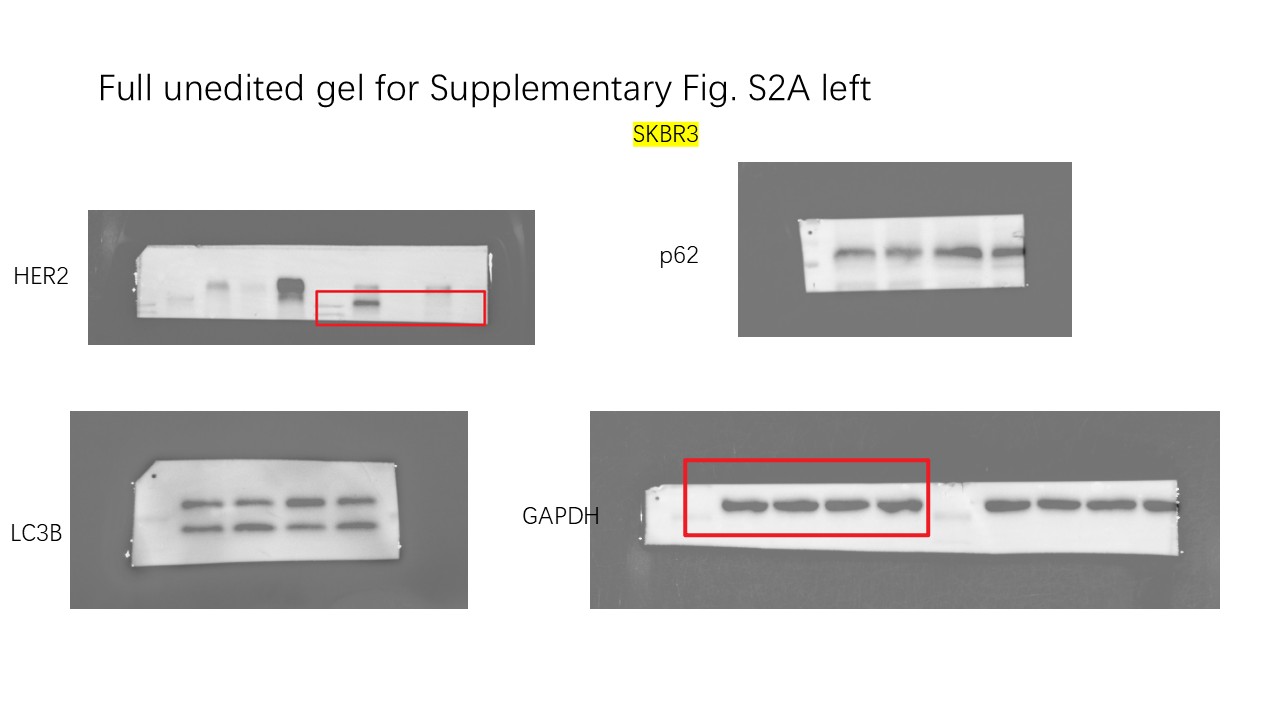


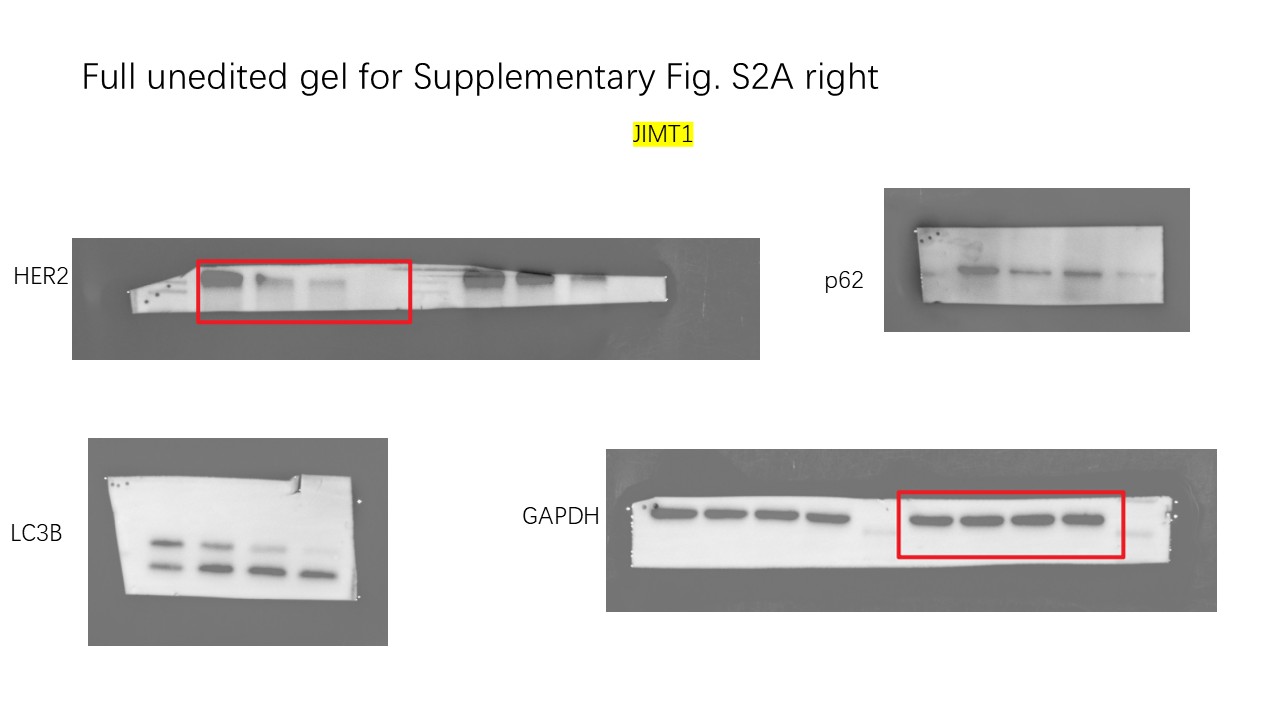


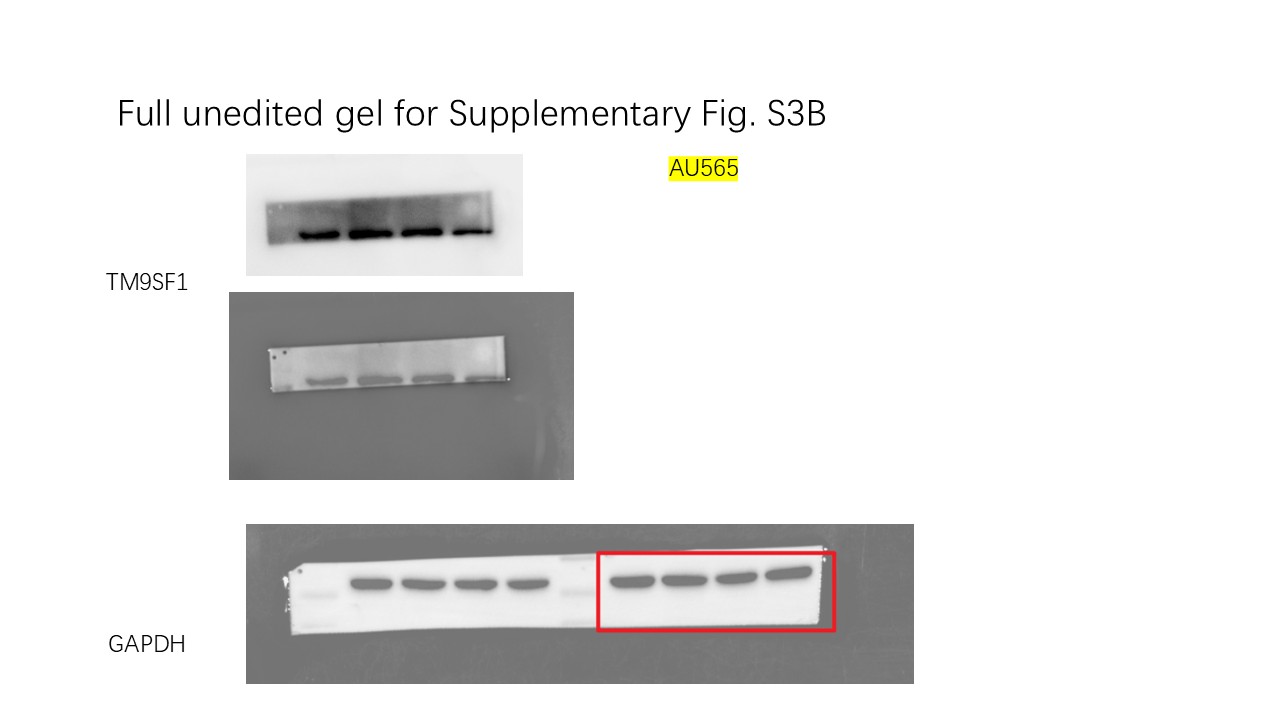


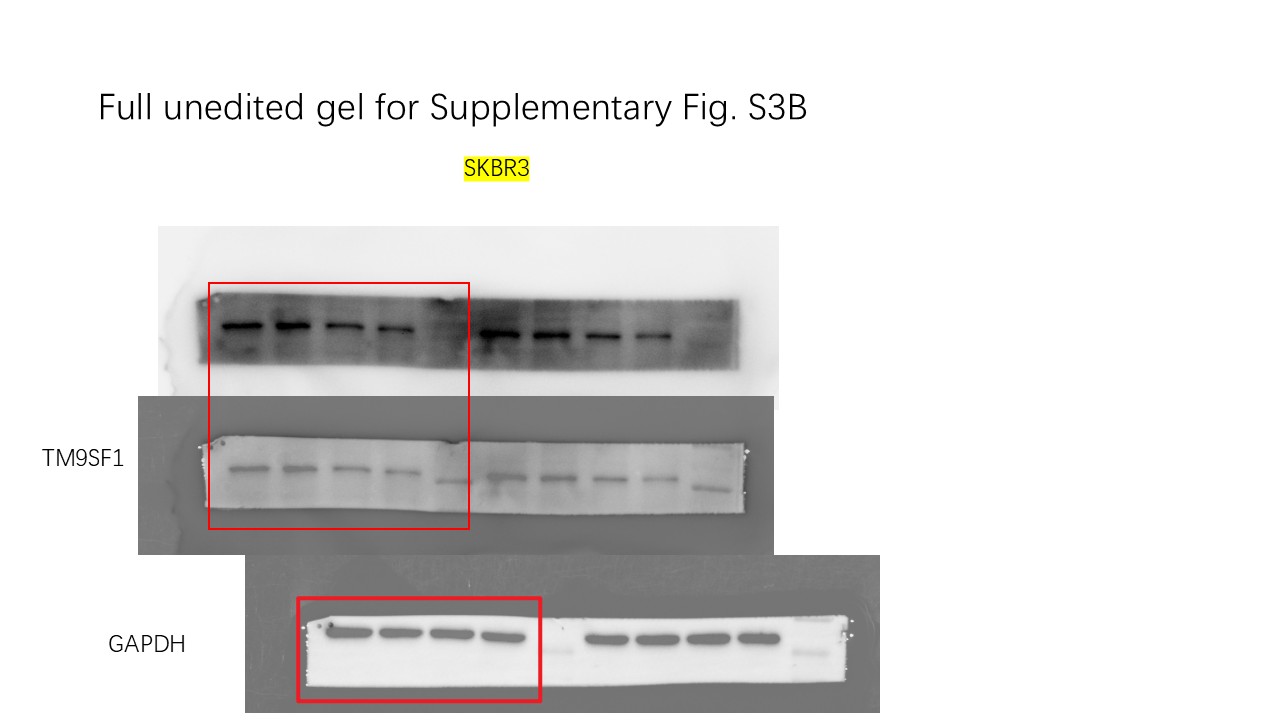


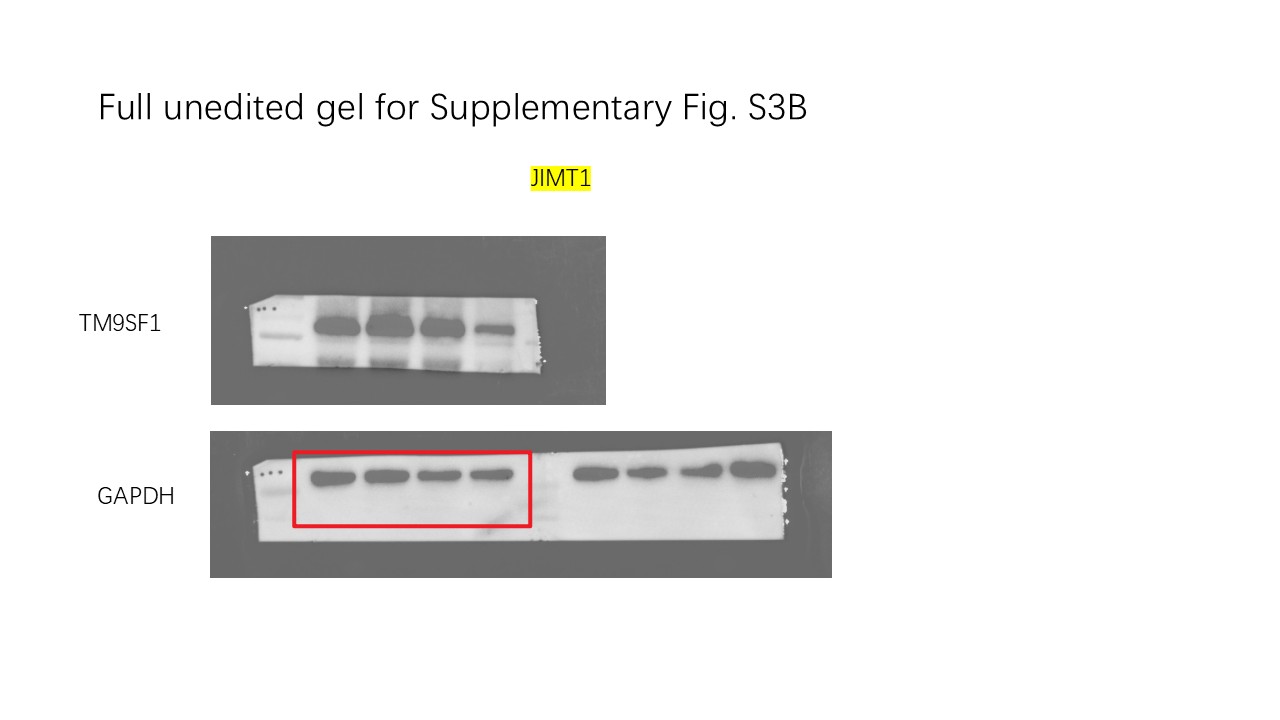


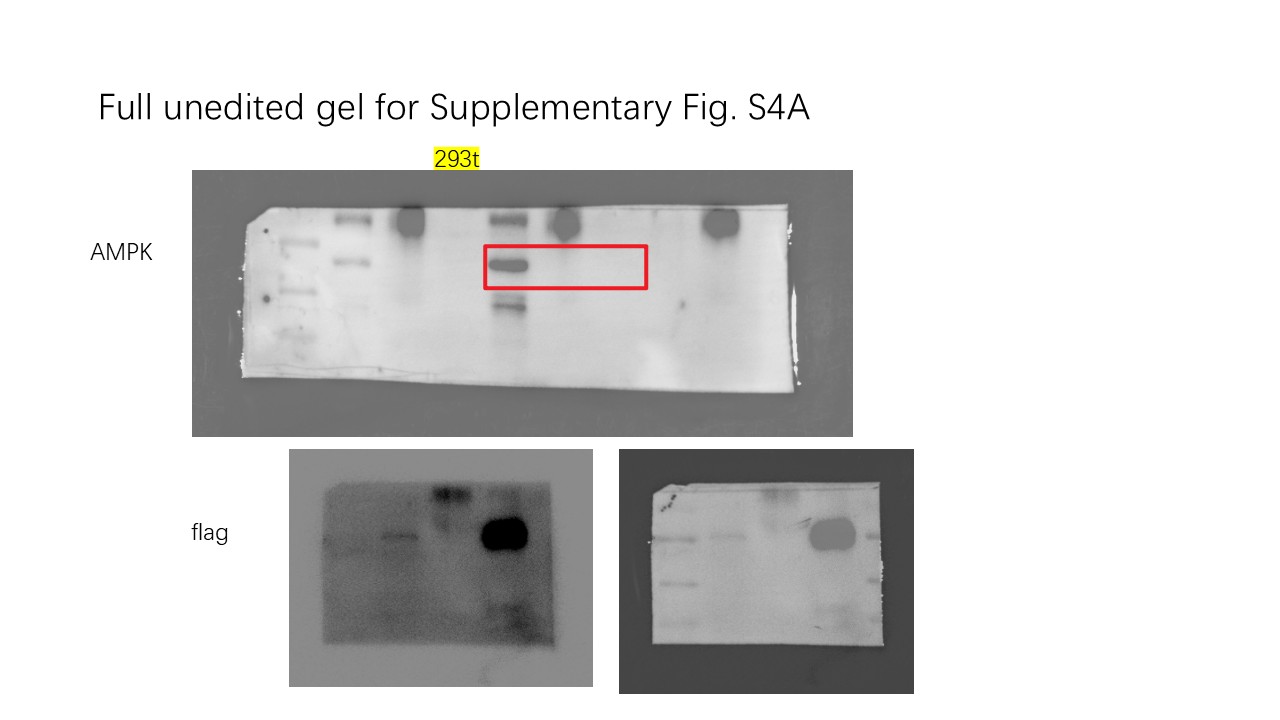

Supplement: Supplementary file 1 — Supplementary Information [file 41419_2025_8093_MOESM1_ESM.docx]
